# Boosting carrier mobility and stability in indium–zinc–tin oxide thin-film transistors through controlled crystallization

**DOI:** 10.1038/s41598-020-76046-w

**Published:** 2020-11-02

**Authors:** Nuri On, Bo Kyoung Kim, Yerin Kim, Eun Hyun Kim, Jun Hyung Lim, Hideo Hosono, Junghwan Kim, Hoichang Yang, Jae Kyeong Jeong

**Affiliations:** 1 Department of Electronic Engineering, Hanyang University, Seoul, 133-791 Republic of Korea; 2 Department of Chemical Engineering, Inha University, Incheon, 22212 South Korea; 3 R&D Center, Samsung Display, Yongin, 17113 South Korea; 4 Materials Research Center for Element Strategy, Tokyo Institute of Technology, Yokohama, 226-8503 Japan

**Keywords:** Materials for devices, Electrical and electronic engineering, Electronics, photonics and device physics

## Abstract

We investigated the effect of film thickness (geometrical confinement) on the structural evolution of sputtered indium-zinc-tin oxide (IZTO) films as high mobility n-channel semiconducting layers during post-treatment at different annealing temperatures ranging from 350 to 700 °C. Different thicknesses result in IZTO films containing versatile phases, such as amorphous, low-, and high-crystalline structures even after annealing at 700 °C. A 19-nm-thick IZTO film clearly showed a phase transformation from initially amorphous to polycrystalline bixbyite structures, while the ultra-thin film (5 nm) still maintained an amorphous phase. Transistors including amorphous and low crystalline IZTO films fabricated at 350 and 700 °C show reasonable carrier mobility (*µ*_*FE*_) and on/off current ratio (*I*_*ON/OFF*_) values of 22.4–35.9 cm^2^ V^−1^ s^−1^ and 1.0–4.0 × 10^8^, respectively. However, their device instabilities against positive/negative gate bias stresses (PBS/NBS) are unacceptable, originating from unsaturated bonding and disordered sites in the metal oxide films. In contrast, the 19-nm-thick annealed IZTO films included highly-crystalline, 2D spherulitic crystallites and fewer grain boundaries. These films show the highest *µ*_*FE*_ value of 39.2 cm^2^ V^−1^ s^−1^ in the transistor as well as an excellent I_ON/OFF_ value of 9.7 × 10^8^. Simultaneously, the PBS/NBS stability of the resulting transistor is significantly improved under the same stress condition. This promising superior performance is attributed to the crystallization-induced lattice ordering, as determined by highly-crystalline structures and the associated formation of discrete donor levels (~ 0.31 eV) below the conduction band edge.

## Introduction

Amorphous indium gallium zinc oxide (*a*-IGZO) has been used as a semiconducting channel material in active-matrix thin-film transistors (TFTs) for high-resolution liquid crystal and large organic light-emitting displays (OLEDs) since its discovery by Hosono and co-workers in 2004^1^. Its attractive properties, such as high carrier mobility (*µ*_*FE*_) of > 10 cm^2^ V^−1^ s^−1^, superior uniformity, ultra-low leakage current (< 1 pA), low temperature processing and low cost fabrication have facilitated rapid commercialization of pixel switchers and drivers in advanced display devices. The ever-increasing demand for larger screens, higher pixel resolution and higher frame rates for immersive and impressive image quality, however, have driven researchers to identify new routes to make much faster transistors. Thus, various semiconducting metal oxide materials such as indium zinc oxide (IZO), zinc tin oxide (ZTO), indium gallium tin oxide (IGTO) and indium zinc tin oxide (IZTO) have been investigated as alternatives of IGZO^1–6^. Among them, an amorphous IZTO (*a*-IZTO) was identified to have a high *µ*_*FE*_ value of ≥ 30 cm^2^ V^−1^ s^−1^ in field-effect transistors (FETs). The synergic intercalation between the 5 s orbitals of Sn^4+^ and In^3+^ cations provides an efficient percolation conduction pathway, leading to low effective electron mass and enhanced carrier mobility in the resulting FETs^7^. However, the device stability is a critical factor in actual implementation and remains an issue because many gap states in the forbidden bandgap of *a*-IZTO are responsible for the bias-induced carrier trapping and the resulting threshold voltage (*V*_*TH*_) instability^8,9^.

Recently, the semiconducting oxides have been also researched as an alternative channel material for vertical NAND (V-NAND) flash memory devices^10–14^. The low mobility (≤ 10 cm^2^/Vs) and non-negligible leakage current of polycrystalline Si thin-film transistors adopted in current V-NAND still limit the maximum number of vertically stackable layer. The high mobility (≥ 30 cm^2^ V^−1^ s^−1^) and excellent low leakage current of oxide FETs can mitigate these issues of polycrystalline Si FETs. Thus, the various charge trap stacks such as SiO_2_/Si_3_N_4_/SiO_2_, Al_2_O_3_/HfO_2_/Al_2_O_3_ in conjunction with amorphous IGZO channel layer have been studied at a relatively low temperature (≤ 400 °C)^15–17^. However, the high-temperature processability of IZTO channel layer in terms of structural and electrical properties has not been investigated yet even though the maximum process temperature of current V-NAND is larger than 700 °C. Among the structural phases of semiconducting oxides, most of the research has focused on amorphous phases, which can provide the inherent benefits of uniform mobility and *V*_*TH*_, leading to beneficial switching characteristics. However, amorphous relaxation causes many defects, such as bond length/angular disordering, unsaturated coordination, dangling bonds, etc. Bistability sites are responsible for severe threshold voltage (*V*_*TH*_) instability^18–24^. In this regard, polycrystalline oxide semiconductor-based transistors have been studied to achieve highly stable electronic devices. A single crystal-like InGaO_3_(ZnO)_5_ (IGZO) layer yields a high *µ*_*FE*_ value of approximately 80 cm^2^ V^−1^ s^−1^ in the FET, but it can be formed at an extremely high annealing temperature of 1400 °C for 30 min^25^. An as-deposited *a*-IGZO film is known to crystallize during annealing at 600 to 700°C^26–29^.

In our previous work, metal-induced crystallization reduced the initiation crystallization temperature of IGZO by up to about 300 °C, and the resulting crystalline IGZO layer showed an improved *µ*_*FE*_ value of ~ 54 cm^2^ V^−1^ s^−1^ in FET, 3 times greater than that of *a*-IGZO one^30^. The result clearly suggests that the electrical properties of the semiconducting oxides were promoted by reducing the scattering of charge carriers in the semiconducting layer. However, in-depth understanding of the crystal grain and grain boundary *(GB)* distribution was still lacking because the top metal capping layer covered the structural form of the buried IGZO crystallites. During the crystallization, two- or three-dimensional *GB* defects inevitably occurred, and they can act as charge traps or electrically inactive sites in the crystalline oxide semiconductor systems. It is known that the *GB* defects in Si-based FETs trap charges and form a Schottky barrier, which degrades the carrier mobility^31–33^. However, in metal oxide semiconductor systems, the *GB*-driven trap behavior is an interesting basic subject of material science and is important for potential industrial applications such as display, memory, logic and sensor devices, but it has not been systematically investigated.

Here, we investigated the evolution of microstructures in IZTO films of different thickness through high-temperature annealing at 700 °C. By varying film thickness and annealing time, the structural phases in the annealed IZTO films changed from amorphous to partially crystallized to well crystallized microstructures. The topology and phase identification of the IZTO films were examined to understand how these microstructures affect the electrical performance of the resulting IZTO FETs. The reason for selecting high temperature annealing at 700 °C was that this temperature was enough to induce controllable crystallization from as-deposited *a*-IZTO films. For semiconductor devices on Si substrates such as DRAM, NAND and logic devices, this thermal budget is generally acceptable due to the high melting temperature (approximately 1410 °C) of silicon. An in-depth understanding of the lattice ordering, grain size, and *GB* in the thickness-controlled IZTO phases could provide insight into the feasibility of polycrystalline oxide semiconductors for highly stable display backplanes and/or three-dimensional silicon-based electronics. An optimized 19-nm-thick IZTO film including a highly crystalline and less *GB* structure showed a high *µ*_*FE*_ value of 39.2 cm^2^V^−1^ s^−1^ in the transistor, as well as an excellent *I*_*ON/OFF*_ value of 9.7 × 10^8^. The positive/negative gate bias stress (PBS/NBS) stability of the resulting transistors was considerably improved under the same stress conditions. Our major finding was that the optimized polycrystalline IZTO FETs exhibited better electrical performance than the corresponding amorphous and partially crystallized IZTO FETs, suggesting that the lattice ordering and *GB* minimization through intentional crystallization can be an alternative approach to make highly stable, high performance oxide backplanes or electronics.

## Results

### Optimal annealing of IZTO films

First, we investigated the effect of annealing temperature (*T*_*A*_) on the electrical properties of the IZTO films for high performance FETs. As-prepared IZTO films with a thickness of 19 nm were annealed at 350, 600, and 700 °C for 1 h, respectively. Electrical properties of these IZTO FETs were summarized in the Supporting Information (SI, see Figure S1 and Table S1). The 350 °C-annealed IZTO FETs showed an average *µ*_*FE*_ value of 35.9 cm^2^ V^−1^ s^−1^, a *SS* of 0.24 V decade^−1^ and a *V*_*TH*_ of − 0.41 V. However, a serious decrease in current modulation capacity (referred to as on and off current ratio, *I*_*ON/OFF*_) was observed for the 600 °C-annealed IZTO FETs. This degradation is mainly attributed to the huge formation of oxygen vacancy (*V*_*O*_) defects with a donor character (see the O *1 s* X-ray photoelectron spectra in Figure S2) where the high free carrier density (> 10^19^ cm^−3^, determined from Hall effect measurement) in the 600 °C-annealed IZTO film makes it difficult to deplete the resulting FET device under the negative gate voltage region^34–37^. Interestingly, the 700 °C-annealed films exhibited optimized transfer characteristics: an average *µ*_*FE*_ value of 39.7 cm^2^ V^−1^ s^−1^, a *SS* of 0.26 V decade^−1^, and a *V*_*TH*_ of − 0.21 V.

XRD patterns of the 19 nm-thick IZTO films annealed at different *T*_*A*_s showed that IZTO films annealed even at 600 °C did not show any clear X-ray peaks, suggesting that the films were still amorphous phases. After annealing at either 650 or 700 °C, the annealed IZTO films clearly showed X-ray diffraction peaks. Specifically, the 700 °C sample showed intense X-ray diffraction peaks, originating from crystal grains (Figure S3). This indicates that the IZTO films consisted of crystallites (this will be discussed later). The depth profiles of hydrogen for three IZTO films, which is known to be a shallow donor, were compared by TOF–SIMS analysis (see Figure S4)^38–42^. The IZTO films annealed at 600 and 700° had the smaller hydrogen concentration than that at 350 °C, which suggests that the simple resistor-like behavior of 600 °C-annealed IZTO FETs cannot be explained by the hydrogen doping effect. Interestingly, crystallization due to minimization of thermodynamic Gibbs free energy during annealing at 700 °C should enhance the phase ordering and crystallinity in the resulting IZTO film, resulting in excellent electrical properties in FETs. It can be inferred that the reduction in V_O_ concentration and high semiconducting functionality for the 700 °C-annealed IZTO film would be related to its crystallization.

### Phase transition of in-doped ZTO films: thickness and annealing time effects

**Figure 1:**
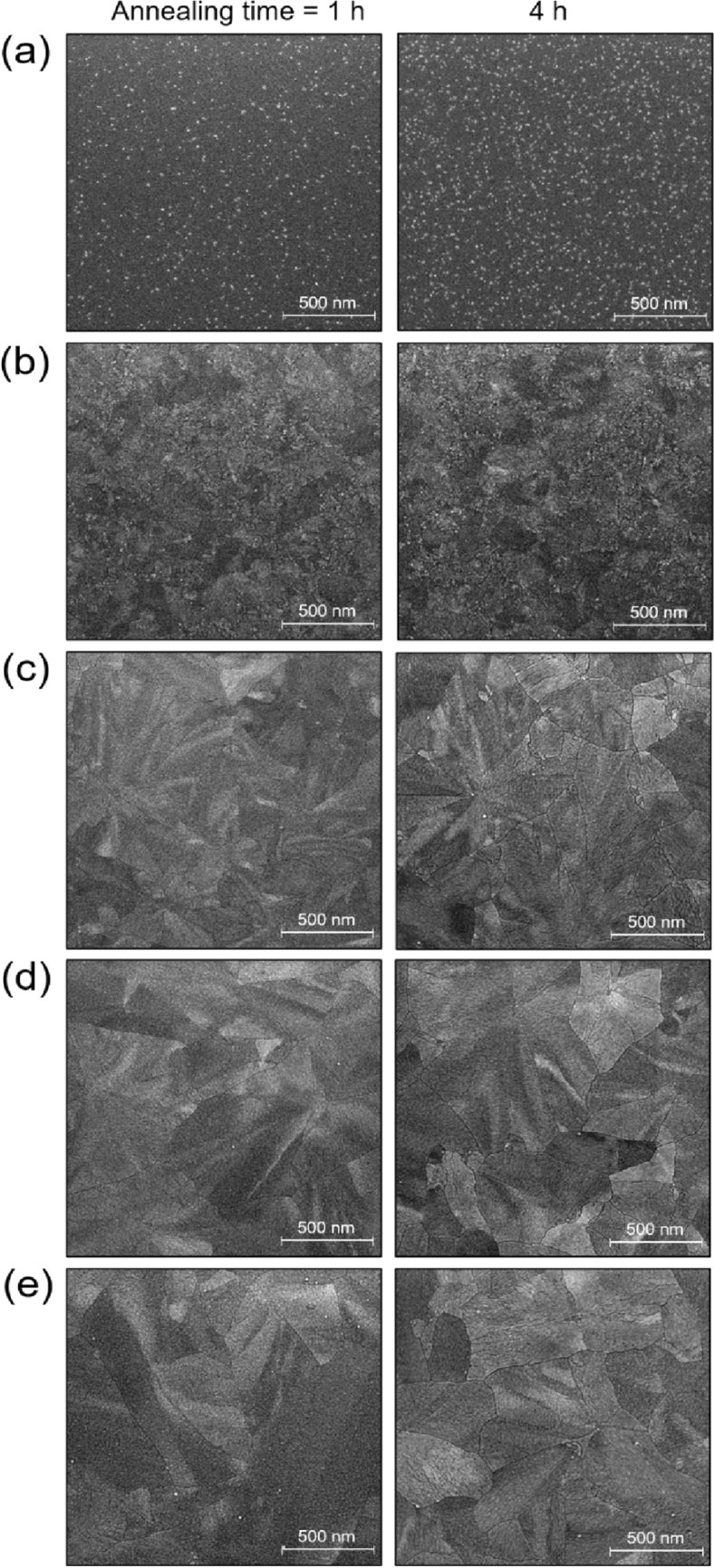
SEM morphologies of IZTO films of different thickness after annealing at 700 °C for 1 and 4 h: (**a**) 5, (**b**) 10, (**c**) 19, (**d**) 30, and (**e**) 50 nm.

It is known that the phase transition behavior of crystallizable metal oxide materials is significantly affected by film thickness (i.e., geometrical confinement) as well as annealing time and *T*_*A*_^43–45^. IZTO films of different thicknesses of 5, 10, 19, 30, and 50 nm were sputtered on SiO_2_ substrates and annealed at 700 °C for 1, 2 or 4 h. Figure 1 shows typical SEM morphologies of the 700 °C-annealed IZTO films showing the structural evolution from particle-like, sheaf-like, to spherulitic grains as a function of the annealing time and thickness. The 5-nm-thick IZTO films showed only small nano-grains with diameters of 10–20 nm because crystallites were difficult to grow in a very confined geometry. As the film became thicker, the featured domains became interconnected and larger laterally. The partially-crystalline 10-nm-thick films showed embedded sheaf-like grains, and thicker films (above 10 nm) contained 2D spherulitic crystallites interconnected with each other. Also, the longer annealing time seemed to increase the *GB* defects. As shown in Fig. 1, SEM morphologies of all the 4 h-annealed samples showed much clearer physical gaps between these crystallites compared to those annealed for shorter times (also see Figure S5). The discernible structure variations in the 700 °C-annealed IZTO films were expected to affect the electrical properties of the resulting FETs.

**Figure 2:**
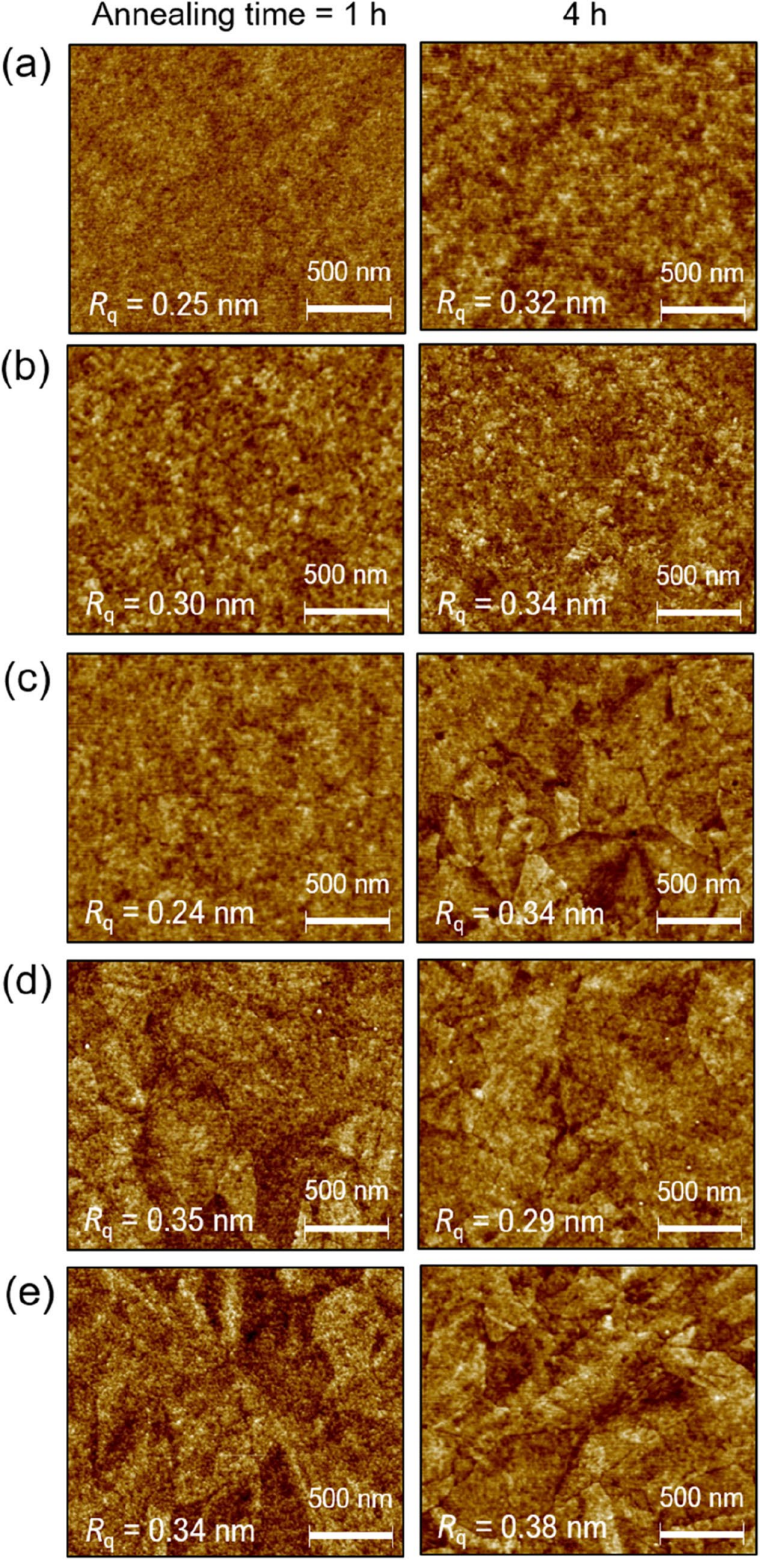
AFM topographies of IZTO films of different thickness after annealing at 700 °C for 1 and 4 h: (**a**) 5, (**b**) 10, (**c**) 19, (**d**) 30, and (**e**) 50 nm. AFM topographies for the IZTO films annealed at 700 °C for 2 h is shown in Figure S7.

The *GB* defect sites in the annealed IZTO films were further investigated using AFM. Figure 2 shows AFM topographies of IZTO films of different thickness on SiO_2_ substrates after annealing at 700 °C for 1 and 4 h. As the film thickness increased, the AFM topographies showed typical particle-like, sheaf-like, and spherulitic grains. The morphological changes of the grains were similar to the trend observed in the SEM results. Nanoparticle-like grains in the 5 and 10-nm-thick films produced smooth film surfaces with a surface roughness (referred to as root mean square roughness, *R*_*q*_) value of 0.25–0.34 nm. Also, sheaf-like and spherulitic grains were grown in a 2D shaped structure rather than a 3D one due to the film thickness limitation. The resulting *R*_*q*_ values varied from 0.24 to 0.38 nm, depending on the grain morphologies. Interestingly, the 19-nm-thick IZTO film annealed for 1 h showed the lowest *R*_*q*_ value of 0.24 nm, suggesting that the 2D spherulites were well interconnected with less defects, suggesting that the charge transfer along these crystallites could be less degraded.

**Figure 3:**
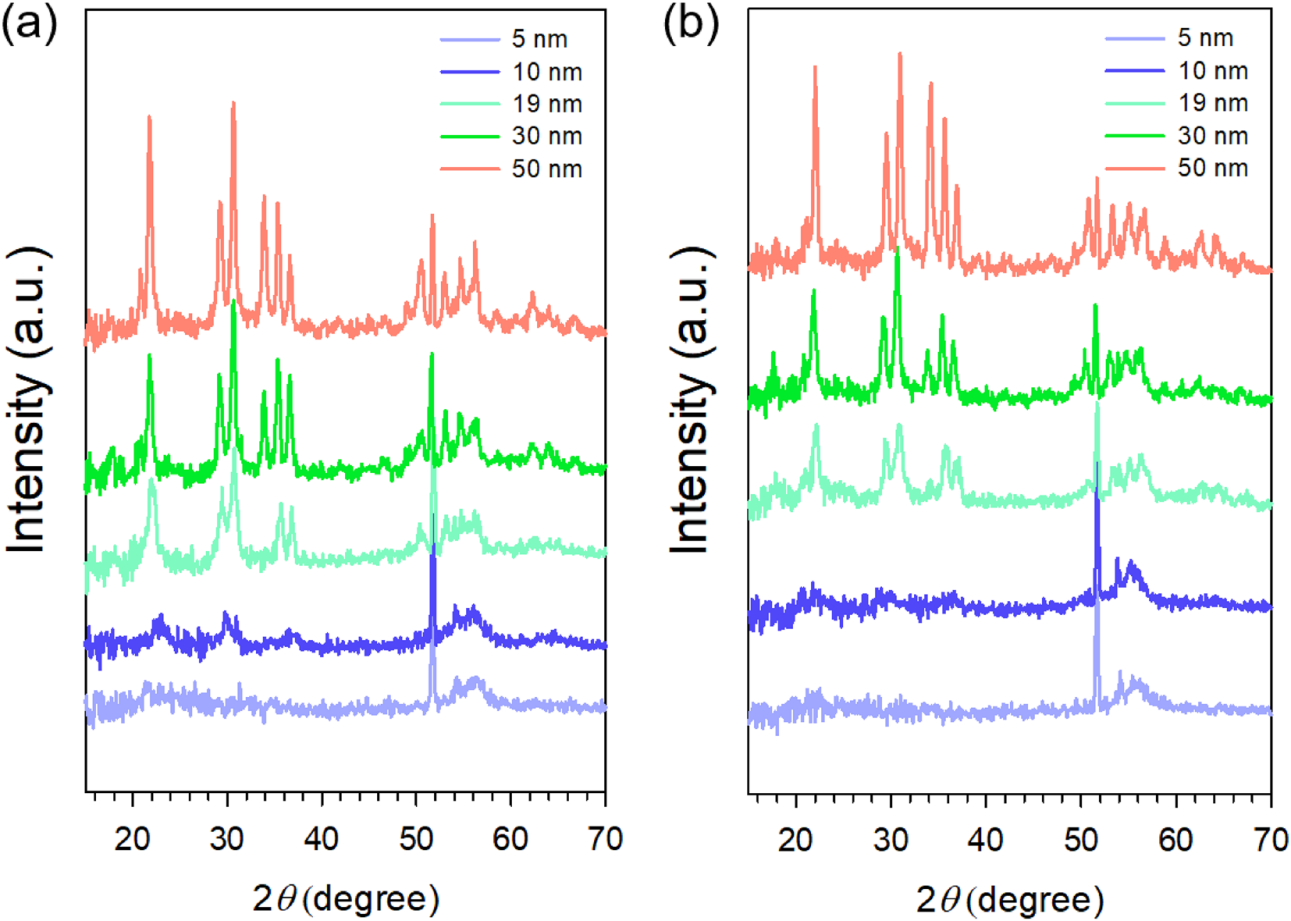
1D X-ray diffraction patterns of IZTO films of different thickness annealed at 700 °C for (**a**) 1 and (**b**) 4 h (the wavelength of X-ray was 1.54056 Å). The 1D X-ray diffraction pattern for the IZTO films at 700 °C for 2 h is shown in Figure S8.

**Figure 4:**
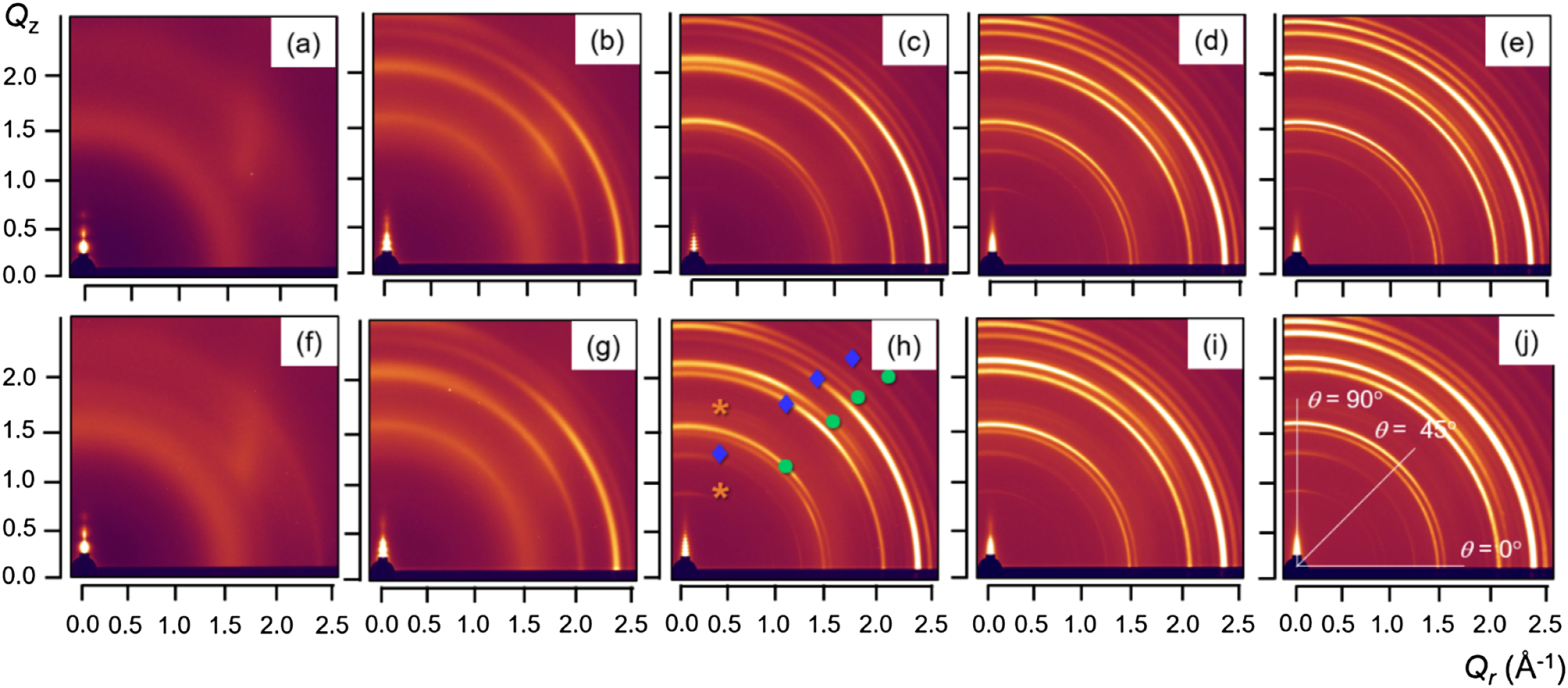
2D GIXD patterns of IZTO films of different thickness after annealing at 700 °C for (**a**–**e**) 1 and (**f**–**j**) 4 h; (**a**,**f**) 5, (**b**,**g**) 10, (**c**,**h**) 19, (**d**,**i**) 30, (**e**,**j**) 50 nm (the orange-, blue-, and green-colored symbol-marked peaks in h represent typical peaks of SnO_2_, Zn_2_SnO_4_, and In_2_O_3_, respectively). 2D GIXD patterns of IZTO films of different thickness after annealing at 700 °C for 2 h are also shown in Figure S10.

Figure 3 shows typical 1D GIXD profiles of IZTO films of different thickness annealed at 700 °C for 1 and 4 h, respectively. First, the 5-nm-thick IZTO films did not show any X-ray diffraction peaks even after annealing at 700 °C for 4 h. The amorphous nature of the 5-nm-thick IZTO film was also confirmed by TEM analysis (see Figure S6). However, the relatively thicker films showed X-ray diffraction peaks in the 2θ range of 20–60°. The peak intensities tended to increase with an increase in *t*_*s*_ and were less dependent on annealing time (Fig. 3a,b). The 10-nm-thick IZTO film showed weak X-ray diffraction peaks, expecting a less ordered and small crystal phase, while each X-ray diffraction peak was clearly indicated above 10 nm. As shown in Fig. 3, typical X-ray profiles of these 700 °C-annealed IZTO films showed diffraction peak series at 2θ = 21.65, 29.50, 30.80, 34.20, 35.58, 36.91, 37.0, and 51.10°, which would be indexed as those of typical (*hkl*) crystal planes, respectively, in spinel Zn_2_SnO_4_ and cubic bixbyite In_2_O_3_ (c-In_2_O_3_) phases (see Figure S9 and Table S2 in SI)^36,46–49^, suggesting that each Zn_2_SnO_4_ and In_2_O_3_ crystallite seemed to be simultaneously developed from the initially *a*-IZTO films during annealing at 700°C^50–54^. In addition, the existence of SnO_2_ crystallites in these films was found from synchrotron-based high resolution GIXD measurements performed for the IZTO films (Fig. 4).

**Figure 5:**
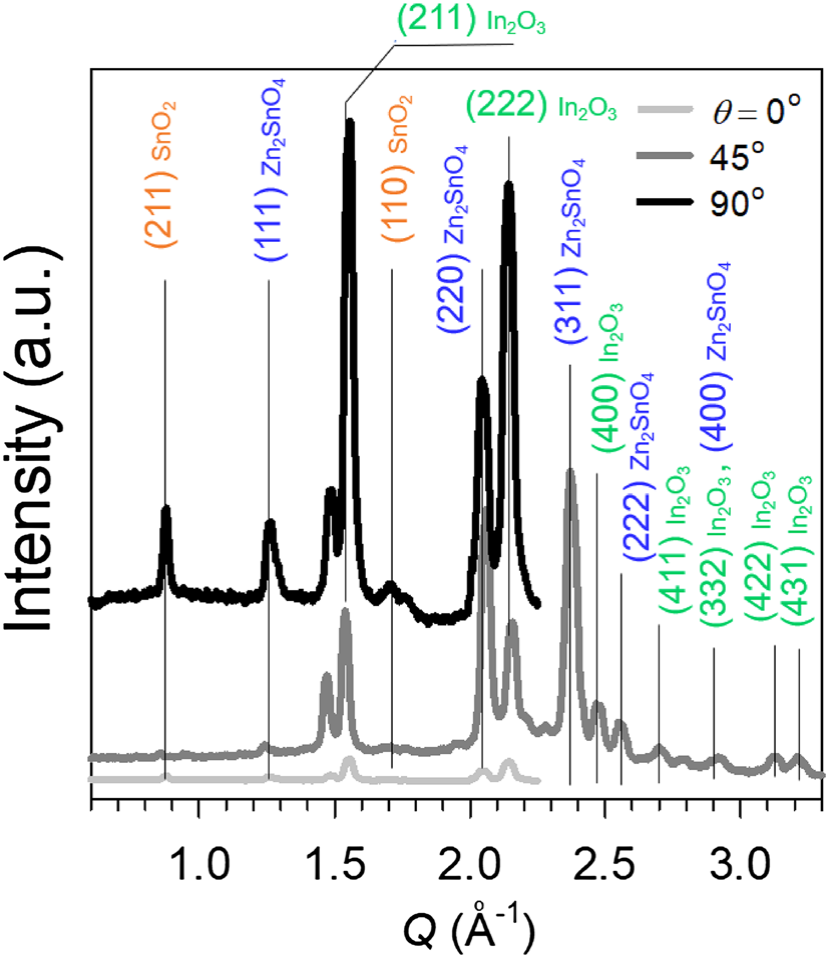
1D GIXD patterns of the 50-nm-thick IZTO film (annealed at 700 °C for 4 h) extracted along a tilting angle (*θ*) = 0, 45, and 90° from the corresponding 2D GIXD pattern.

Similar to the conventional GIXD results, the 5-nm-thick IZTO films did not show any X-ray diffraction peaks (except for the X-ray reflectivity profiles along the *Q*_*z*_ axis) even after annealing at 700 °C for 4 h. As shown in Fig. 4b,g, however, 2D GIXD patterns of the 10-nm-thick annealed films showed anisotropic (not isotropic or Debye) X-ray reflection peaks at *Q* = 1.540, 2.060, 2.372, and 2.475 Å. The initial X-ray reflection peaks appeared to be sharper and more intensely separated as film thickness increased. Also, the 2D GIXD patterns of the 19-, 30-, and 50-nm-thick IZTO films showed two additional peaks at 0.879 and 1.263 Å, as well as others at high *Q* values of above 2.0 Å. As the film thickness increased, the resulting X-ray reflections were broadly scattered. Specifically, 2D GIXD patterns of the 50-nm-thick IZTO film showed Debye ring-like patterns (Fig. 4j). X-ray reflections for both the conventional and synchrotron-based measurements were found to be related to three crystalline phases including SnO_2_, In_2_O_3_, and Zn_2_SnO_4_ crystallites. Figure 5 represents 1D X-ray profiles extracted along the different tilting angle (*θ*) values of 0, 45, and 90° with respect to the substrate for the 2D GIXD patterns of the 50-nm-thick IZTO film annealed at 700 °C for 4 h. The data indicate that the polycrystalline structure is not random but has a certain degree of preferential orientation.

**Figure 6:**
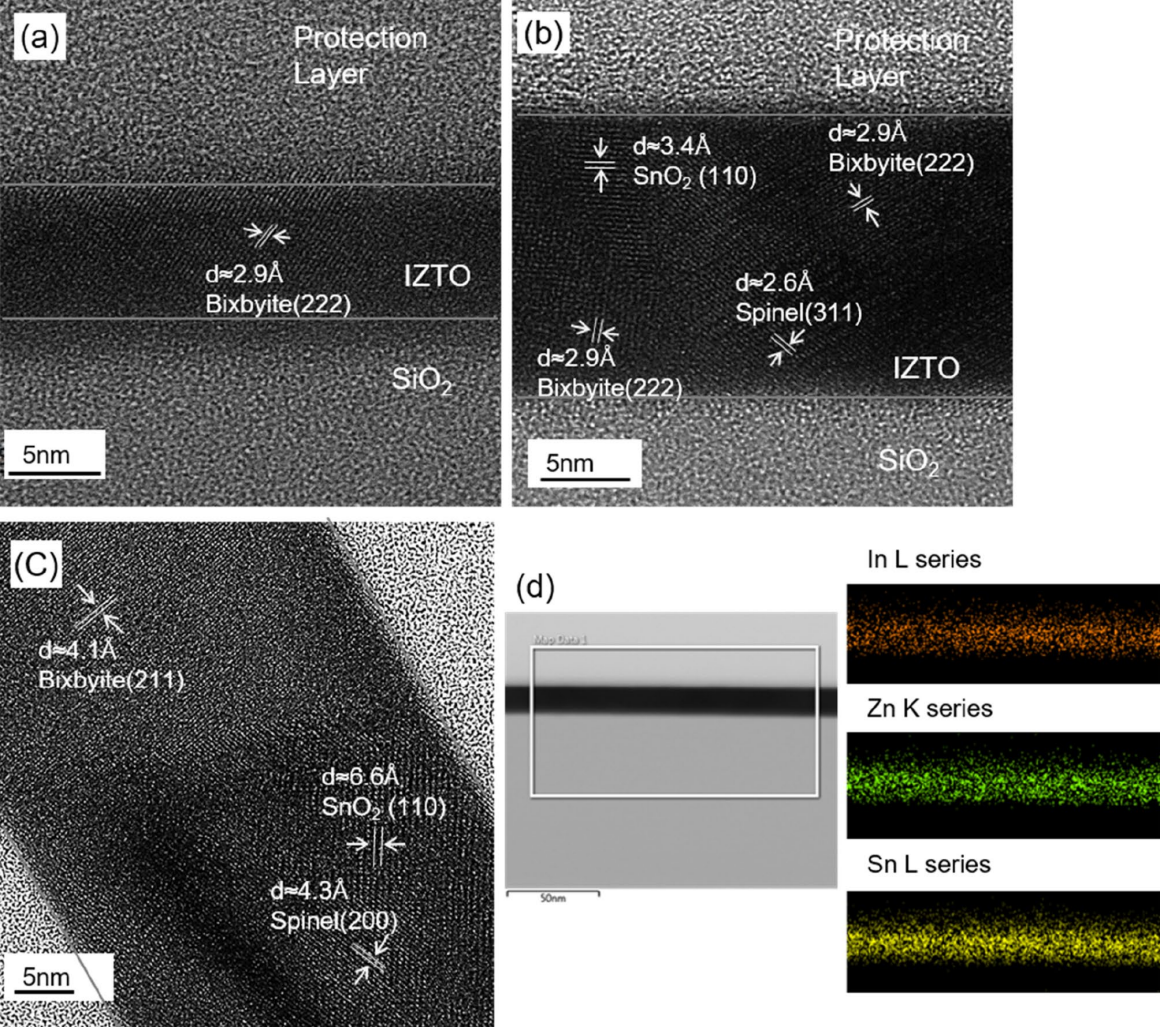
(**a**–**c**) Cross-sectional TEM images of 10, 19 and 50-nm-thick IZTO films. (**d**) Elemental mapping of 19-nm-thick IZTO film.

Figure 6 shows the cross-sectional TEM morphologies, electron diffraction (ED) patterns, and element mapping images of the 10, 19, and 50-nm-thick IZTO films after annealing at 700 °C for 1 h. First, it was found that the 10-nm-thick film was partially crystalline as determined by the diffused ED ring pattern in a sectioned film area (see Figure S11). However, the crystal regions clearly showed aligned planes with a domain-spacing (*d*-spacing) of about 2.90 Å, corresponding to that of *d*_222_ in a bixbyite In_2_O_3_ crystallite. In contrast, the 19-nm-thick IZTO film contained fully-occupied crystallites, where highly-ordered crystal planes also had a *d*-spacing of 2.90 Å, which was attributed to *d*_222_ in a bixbyite In_2_O_3_ crystallite. Lattice images assignable to the spinel Zn_2_SnO_4_ crystallite were observed in other regions of 19-nm-thick IZTO films (data shown in Figure S12). TEM morphologies revealed additional crystal structures with *d*-spacings of 2.60 Å, which can be indexed as (311) of Zn_2_SnO_4_ crystallites. The scanning TEM analysis clearly shows the dispersive distribution of In, Zn, and Sn cations without discernible separation features. This is rather unexpected because the co-existence of three phases of SnO_2_, In_2_O_3_ and Zn_2_Sn_1_O_4_ with finite crystalline size should result in spatial non-uniformity in terms of In, Zn and Sn cations. This indicates that the chemical formula of the thermodynamically stable bixbyite and spinel phase at high temperature would be In_(2-2x)_Zn_x_Sn_x_O_3_ and Zn_(2-y)_Sn_(1-y)_In_2y_O_4_ rather than pure In_2_O_3_ and Zn_2_SnO_4_, respectively, which is known to be a sub-solid phase relationship in the InO_1.5_–ZnO–SnO_2_ system^50^. According to the ternary phase diagram of InO_1.5_–ZnO–SnO_2_ determined at 1,250 °C, the specific composition In_0.23_Zn_0.37_Sn_0.40_O used in this study has the three equilibrium phases including SnO_2_, a bixbyite solid solution In_(2−2x)_Zn_x_Sn_x_O_3_ (x ≈ 0.4) and a spinel solid solution Zn_(2−y)_Sn_(1−y)_In_2y_O_4_ (y ≈ 0.2)^50^. The extended co-solubility of Zn and Sn in the bixbyite In_2_O_3_ phase is rationalized based on the good size-matching and isovalent nature of the substitution, where two trivalent In cations (In^3+^) are substituted by one divalent Zn^2+^ and one tetravalent Sn^4+^ cation. Likewise, the extended solubility of In in the spinel phase can be explained by the opposite of bixbyite co-substitution; that is, two In^3+^ cations are substituted for one Zn^2+^ and one Sn^4+^ site. The 50-nm-thick IZTO film revealed *d*-spacings of 4.12 and 5.05 Å, and the associated crystalline planes were indexed as (211) of bixbyite In_(2−2x)_Zn_x_Sn_x_O_3_ and (111) of spinel Zn_(2−y)_Sn_(1−y)_In_2y_O_4_ crystallites, respectively (also see Figure S13). Based on the three phase identification of SnO_2_, a bixbyite solid solution In_(2−2x)_Zn_x_Sn_x_O_3_ (x ≈ 0.4) and a spinel solid solution Zn_(2−y)_Sn_(1−y)_In_2y_O_4_ (y ≈ 0.2), their corresponding mole fractions in the 700 °C-annealed IZTO film were estimated to be 0.20, 0.20 and 0.60, respectively, using the lever rule principle in the sub-solid ternary phase diagram. The highest fraction (60%) of spinel solid-solution is consistent with its dominant integrated diffraction peak area in 1D and 2D-GIXD analysis. The average grain size in the vertical direction for the crystalline IZTO films was calculated from the full-width-at-half-maximum (FWHM) using Scherrer’s formula (D = kλ/βcos θ, where D is an average grain size along the vertical direction, k is a Scherrer constant (~ 0.9), λ is the wavelength of the incident X-rays (1.54056 Å), β is the FWHM of the given reflection, and θ is the diffraction angle) (Table S3).

**Figure 7:**
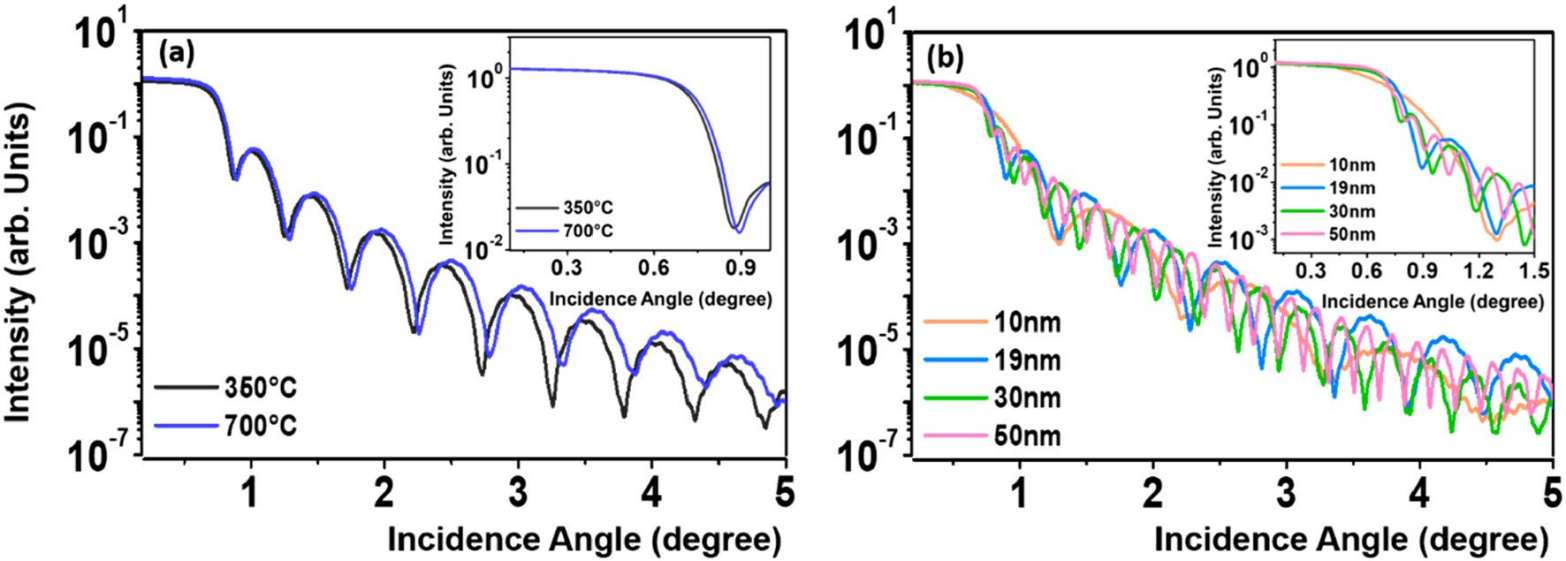
Measured XRR data for the (**a**) 19-nm-thick IZTO films on SiO_2_/Si substrates annealed at 350 and 700 °C and (**b**) 700 °C-annealed IZTO films with different thicknesses of 10, 19, 30, and 50 nm.

The mass densities of the IZTO films were calculated by X-ray reflectivity (XRR) analysis. Figure 7 shows the XRR data for the (a) 19-nm-thick IZTO films on SiO_2_/Si substrates annealed at 350 and 700 °C and (b) 700 °C-annealed IZTO films with different thicknesses of 10, 19, 30, and 50 nm. The critical angle of the total reflection for the 19-nm-thick IZTO film annealed at 700 °C, which is proportional to the electron density for the given film, was larger than that for the same IZTO film annealed at 350 °C, indicating that the mass densification occurs during the crystallization at the elevated *T*_*A*_ of 700 °C. The mass densities (*ρ*_*mass*_) of 19-nm-thick IZTO films at 350 and 700 °C were 6.63 and 6.92 g/cm^3^, respectively, summarized in Table 1. The effect of film thickness on the *ρ*_*mass*_ values for the 700 °C-annealed IZTO films was rather weak as shown in Fig. 7b and Table 1.

**Table 1:** Variations in mass density (*ρ*_*mass*_) of the IZTO films of different thicknesses annealed at different *T*_*AS*_.

Sample conditions	19-nm-thick 350 °C	10-nm-thick 700 °C	19-nm-thick 700 °C	30-nm-thick 700 °C	50-nm-thick 700 °C
*ρ*_*mass*_ (g/cm^3^)	6.63	6.90	6.92	6.94	6.95

### Electrical performance of IZTO-based FETs

**Figure 8:**
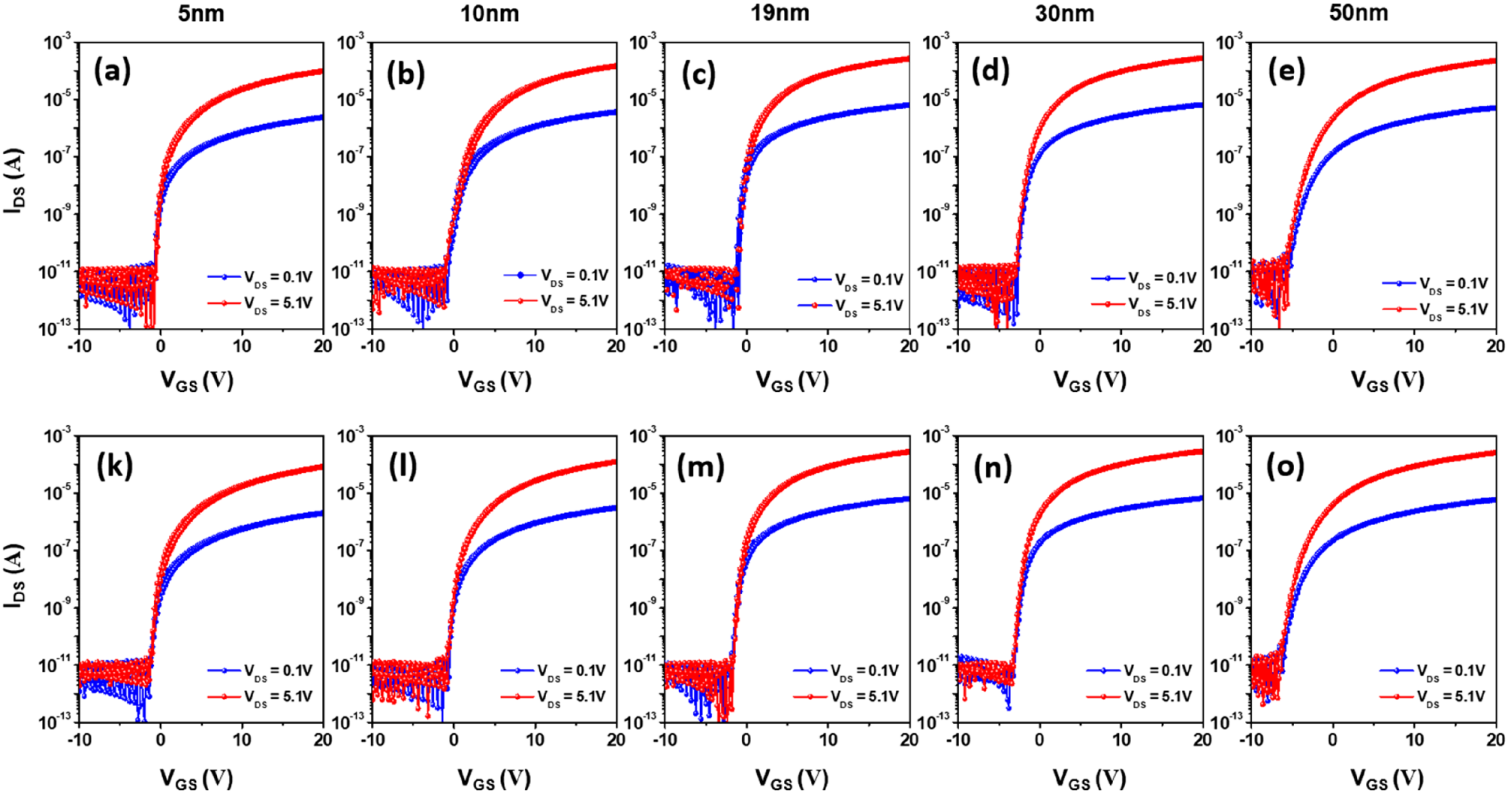
*I*_*DS*_*-V*_*GS*_ transfer curves of FETs including IZTO films of different thickness after annealing at 700 °C for (**a**–**e**) 1 and (**f**–**j**) 4 h: (**a**,**f**) 5, (**b**,**g**) 10, (**c**,**h**) 19, (**d**,**i**) 30, and (**e**,**j**) 50 nm.

Figure 8 shows typical *I*_DS_–*V*_GS_ transfer characteristics of the 700 °C-annealed IZTO FETs with different semiconducting film thicknesses ranging from 5 to 50 nm. All the electrical properties are summarized in Table 2. The device with a 5-nm-thick *a*-IZTO channel annealed for 1 h yielded a reasonable *μ*_FE_ value of 16.6 cm^2^ V^−1^ s^−1^, a *V*_TH_ of 0.33 V and an *I*_ON/OFF_ ratio of 10^8^. The device performance did not vary much with the annealing time, in which times from 1 to 4 h resulted in *μ*_*FE*_ values of 15.8–16.8 cm^2^ V^−1^ s^−1^ and *V*_TH_ values of 0.13–0.33. A similar trend was observed for other thick IZTO FETs (also see Figure S14 and Table S4), so the annealing time effect will not be discussed hereafter. Interestingly, 10-nm-thick IZTO films with partially crystalline structures showed relatively improved *μ*_FE_ values of 22.4–24.2 cm^2^ V^−1^ s^−1^ in FETs, as well as *V*_TH_ values of 0.63–1.00 V and an *I*_ON/OFF_ of ~ 10^8^. Among the polycrystalline IZTO films with percolated spherulites, the thinnest 19-nm-thick film-based FETs showed *µ*_FE_ values of 39.2–39.7 cm^2^ V^−1^ s^−1^, which were 2.4 times greater than those of *a*-IZTO film FETs, resulting from the improved lattice ordering induced during the high temperature crystallization. The lattice ordering allows individual electron waves to be transported along the semiconducting film without energy loss, known as the “coherent scatting mechanism”. However, uncontrolled crystal nucleation and growth carries the risk of forming adverse defects such as lattice-mismatched grain boundaries (*GB*), which may act as undesirable trap sites causing the stretch-out of the sub-threshold drain current region in FETs. Therefore, this trade-off relationship between carrier mobility and trap density should be carefully studied to fully understand the crystallization process.

**Table 2:** Typical electrical parameters of the FETs including IZTO films of different thickness annealed at 700 °C for 1 and 4 h.

IZTO thickness	Annealing time	*µ*_FE_ (cm^2^ V^−1^ s^−1^)	*SS* (V decade^−1^)	*V*_TH_ (V)	*I*_ON/OFF_	*D*_it,max_ (cm^−2^ eV^−1^)	*N*_T, max_ (cm^−3^ eV^−1^)
5 nm	1	16.6 ± 1.7	0.50 ± 0.03	0.3 ± 0.2	9.75 × 10^7^	1.8 × 10^12^	3.7 × 10^18^
	4	15.8 ± 1.8	0.53 ± 0.04	0.1 ± 0.2	9.34 × 10^7^	1.9 × 10^12^	3.9 × 10^18^
10 nm	1	24.2 ± 1.0	0.59 ± 0.09	1.0 ± 0.2	4.00 × 10^8^	2.2 × 10^12^	2.2 × 10^18^
	4	22.4 ± 1.5	0.55 ± 0.08	0.6 ± 0.3	4.04 × 10^8^	2.0 × 10^12^	2.0 × 10^18^
19 nm	1	39.7 ± 2.1	0.26 ± 0.06	− 0.2 ± 0.4	9.70 × 10^8^	9.5 × 10^11^	5.0 × 10^17^
	4	39.7 ± 2.4	0.27 ± 0.07	− 0.7 ± 0.4	9.32 × 10^8^	9.9 × 10^11^	5.2 × 10^17^
30 nm	1	39.7 ± 3.0	0.46 ± 0.09	− 1.3 ± 1.5	9.24 × 10^8^	1.7 × 10^12^	5.6 × 10^17^
	4	39.9 ± 3.4	0.42 ± 0.10	− 2.1 ± 1.4	9.43 × 10^9^	1.5 × 10^12^	5.1 × 10^17^
50 nm	1	34.9 ± 2.0	0.97 ± 0.12	− 4.0 ± 2.3	9.75 × 10^8^	3.6 × 10^12^	7.1 × 10^17^
	4	36.5 ± 2.2	0.97 ± 0.13	− 4.2 ± 2.2	9.86 × 10^8^	3.6 × 10^12^	7.1 × 10^17^

The *SS* value of a given FET device is a fingerprint of the total density of traps, including the fast bulk (*N*_*T*_) and semiconductor-insulator trap (*D*_*it*_), as follows^55^:

1 $$SS = \frac{{qk_{B} T\left( {N_{T} t_{ch} + D_{it} } \right)}}{{C_{i} \log \left( e \right)}}$$

Here q is the electron charge, *k*_*B*_ is Boltzmann’s constant, *T* is the absolute temperature, and *t*_*ch*_ is the channel layer thickness. Note that *N*_*T*_ and *D*_*it*_ values were calculated after the other parameter was set to zero, because these values corresponded to the maximum trap density existing in the system. It was found that the 5-nm-thick a-IZTO channel had *N*_*T*_ and *D*_*it*_ values of 3.66 × 10^18^ eV^−1^ cm^−3^ and 18.3 × 10^11^ eV^−1^ cm^−2^, respectively. The partially-crystalline film (with 10 nm thickness) showed similar values: *N*_*T*_ = 2.16 × 10^18^ eV^−1^ cm^−3^ and *D*_*it*_ = 21.6 × 10^11^ eV^−1^ cm^−2^. As expected, the 19-nm-thick films with fully interconnected crystallites showed the lowest *N*_*T*_ and *D*_*it*_ values of 0.50 × 10^18^ eV^−1^ cm^−3^ and 9.51 × 10^11^ eV^−1^ cm^−2^, suggesting that the improved lattice packing of the large 2D spherulites can compensate for the slow charge-carrier transport behavior expected at GB sites. The smaller *N*_*T*_ value for the crystallized IZTO device can be understood by considering the following fact. The gap states in amorphous or weakly crystallized IZTO semiconductors come from the lattice disorder or under-saturated bonds (*V*_*O*_ or *V*_*M*_), which are responsible for the larger *N*_*T*_ value of their FETs. These completely disappear for the fully crystallized IZTO device. Instead, GB-related trap states are created in the forbidden gap of crystalline IZTO, which is observed in the crystalline FETs. Obviously, this indicates that the effective density of the GB-related trap states in crystallize IZTO is smaller than that of the disordered or under-statured bond induced gap states in the amorphous and partially crystalline IZTO films.

The other promising feature of transistors with the 19-nm-thick IZTO channel layer is the improved switching modulation capability, i.e., *I*_*ON/OFF*_ value of 9.7 × 10^8^, which was greater than those (1.0–4.0 × 10^8^) of amorphous and partially-crystalline IZTO-based FETs. This result suggests that the *GB* defects do not cause an adverse leakage current. It is known that the existence of *GB* defects in the poly-Si FETs is responsible for the notorious increase in leakage current under an off-state biasing condition^56,57^. Some leakage current phenomena such as band-to-band tunneling, thermal/field emission, and impact ionization in the depletion region in these transistors are accelerated by the existence of *GB* traps, which have mid-gap trap states in the forbidden region of the Si semiconductor. In contrast, the excellent off-state current value in the polycrystalline IZTO transistor is related to its wide bandgap nature (> 3.0 eV), which is much larger than that (~ 1.1 eV) of Si. Thus, the energy levels of *GB* defects become deeper in the forbidden band of IZTO semiconductors, making these deeper levels inaccessible by the gate voltage induced Fermi-level sweep. Therefore, the polycrystalline IZTO provides enhanced *µ*_*FE*_, lower *SS*, and excellent *I*_*ON/OFF*_ switching capability in the resulting FETs as compared to their polycrystalline Si counterparts. However, *V*_*TH*_ values for the FETs with a thicker IZTO channel layer (≥ 30 nm) were shifted in the negative direction (i.e. depletion mode), although high *µ*_*FE*_ values of 35.0–39.7 cm^2^ V^−1^ s^−1^ were achieved, comparable to that of the 19-nm-thick film. The *V*_*TH*_ values for all the polycrystalline IZTO-based FETs with 19, 30 and 50 nm-thick IZTO channel layers were -0.21 ± 0.35 (for 19 nm), − 1.57 ± 1.45 (for 30 nm), and − 3.97 ± 2.30 V (for 50 nm), respectively (see Table 2). The negative shift in *V*_*TH*_ values for the n-channel FETs is related to an increase in the *N*_*e*_ of the polycrystalline IZTO films. In fully depleted thin-film transistors, *V*_*TH*_ values can be represented by Eq. (2)^23,58^:

2 $$V_{TH} = V_{o} - \frac{{qN_{e} t_{ch} }}{{C_{i} }} - \frac{{qN_{e} t_{ch}^{2} }}{{2\varepsilon_{o} \varepsilon_{r} }}$$

where *V*_*0*_ is the non-ideality related constant, and *ε*_*o*_ and *ε*_*r*_ are the vacuum permittivity and relative dielectric constant of crystalline IZTO semiconductor, respectively. Non-linear fit between *t*_*ch*_ and *V*_*TH*_ for the polycrystalline IZTO film-based FETs gave an *N*_*e*_ value of approximately 2.4 × 10^17^ cm^−3^ for the 10 to 50 nm IZTO films with polycrystalline structures, which is comparable to those (2.4 (± 0.25) × 10^17^ cm^−3^) from Hall effect measurements. This result strongly suggests that the turn-voltage (or *V*_*TH*_) value negatively shifted as the *t*_*ch*_ value increased, as similarly reported in the *a*-IGZO transistor system^23^. It is also noted that *N*_*T*_ values of the 30- and 50-nm-thick IZTO FETs were 5.1–5.6 × 10^17^ and 7.1 × 10^17^ eV^−1^ cm^−3^, respectively, slightly higher than that of the 19-nm-thich IZTO devices, although their *I*_*ON/OFF*_ values were still high enough at 9.2–9.8 × 10^8^. This result is mainly related to the diverse crystal orientation in the thicker films, where radially-grown 3D crystallites could be formed, and most 2D-shaped spherulites grew in a very confined thin layer (see Figs. 3 and 4). The co-existence of differently shaped and oriented crystallites can increase the *GB*-related trap sites, specifically, with an increase in channel thickness. It should be noted that the similar thickness dependent deterioration in terms of trap density is reported for the CAAC-IGZO TFTs^59^. It underpins the importance of microstructure control in the polycrystalline IZTO channel layer for use in FETs. The control of preferential orientation and single spinel phase via cation composition, thermal annealing and atmosphere etc.are expected to further reduce the bulk trap density and improve the switching properties of the resulting IZTO TFTs.

**Figure 9:**
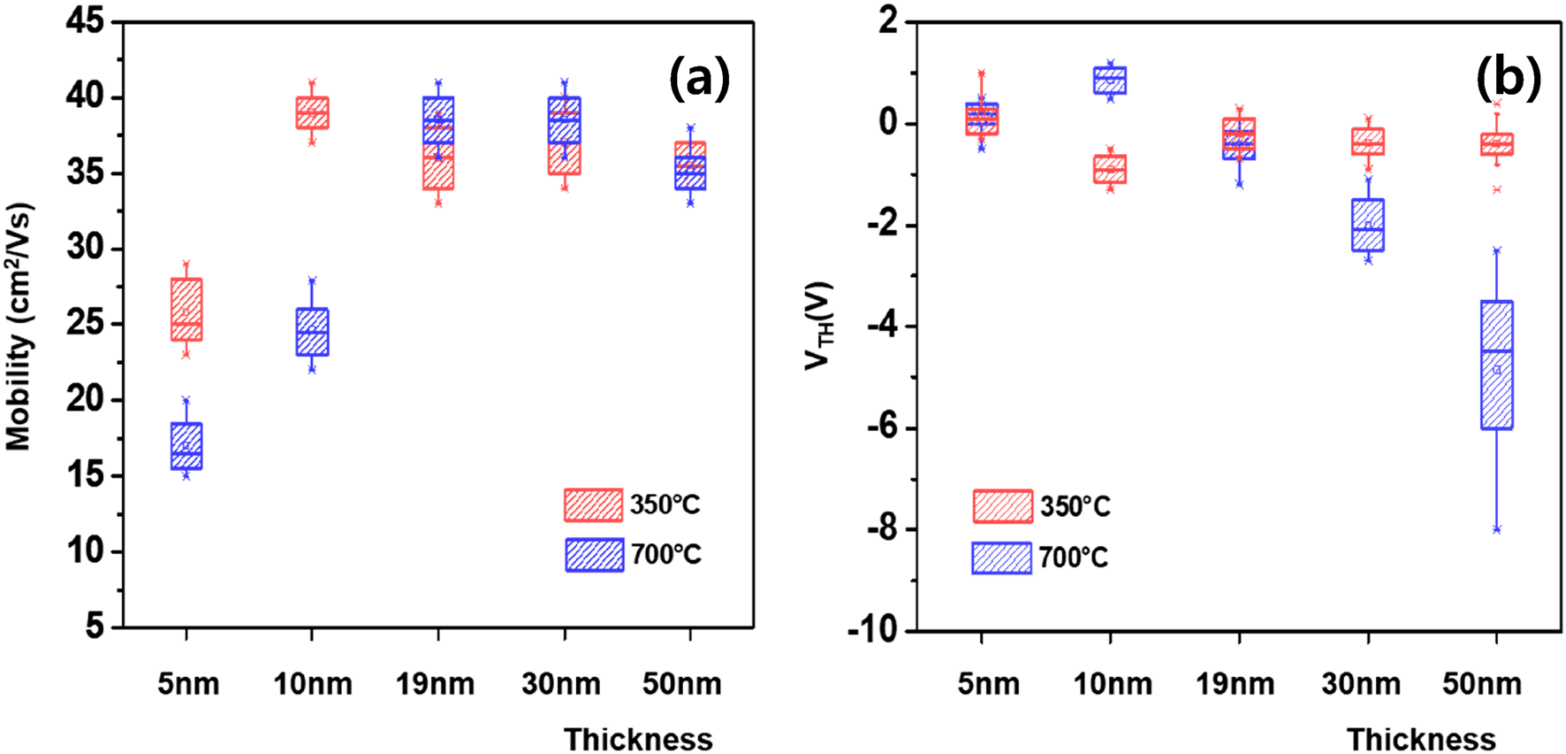
Box plot of the *µ*_*FE*_ and *V*_*TH*_ values for the FETs with 19, 30 and 50-nm-thick IZTO channel layers. The average and standard deviation for the *µ*_*FE*_ and *V*_*TH*_ values were obtained IZTO FETs with different channel thicknesses at *T*_*A*_ = 350 °C are shown in Figure S15 and Table S5.

Figure 9 shows the box plot of *µ*_*FE*_ and *V*_*TH*_ values for the FETs with 5,10, 19, 30 and 50-nm-thick IZTO channel layers. The standard deviation of *µ*_*FE*_ values for the IZTO FETs at *T*_*A*_ = 700 °C was rather independent of the *t*_*ch*_ (Fig. 9a). In contrast, the non-uniformity for the IZTO FETs at *T*_*A*_ = 700 °C in terms of *V*_*TH*_ value became worse with increasing *t*_*ch*_ (≥ 30 nm), whereas a comparable spread of *V*_*TH*_ values was observed for the FETs with a *t*_*ch*_ ≤ 19 nm. To obtain insight into the reason for the *t*_*ch*_ (≥ 30 nm)-dependent *V*_*TH*_ non-uniformity, these statistical data were compared to those for the *a*-IZTO FETs at *T*_*A*_ = 350 °C. For the amorphous phase, there was no strong dependence of *t*_*ch*_ on the spread of *V*_*TH*_ value. Therefore, the negative impact of increasing *t*_*ch*_ (≥ 30 nm) on *V*_*TH*_ distribution should be attributed to the diverse crystal orientation in the thicker film as previously mentioned. This indicates that the thickness of polycrystalline IZTO films (~ 19 nm in this study) should be carefully chosen.

**Figure 10:**
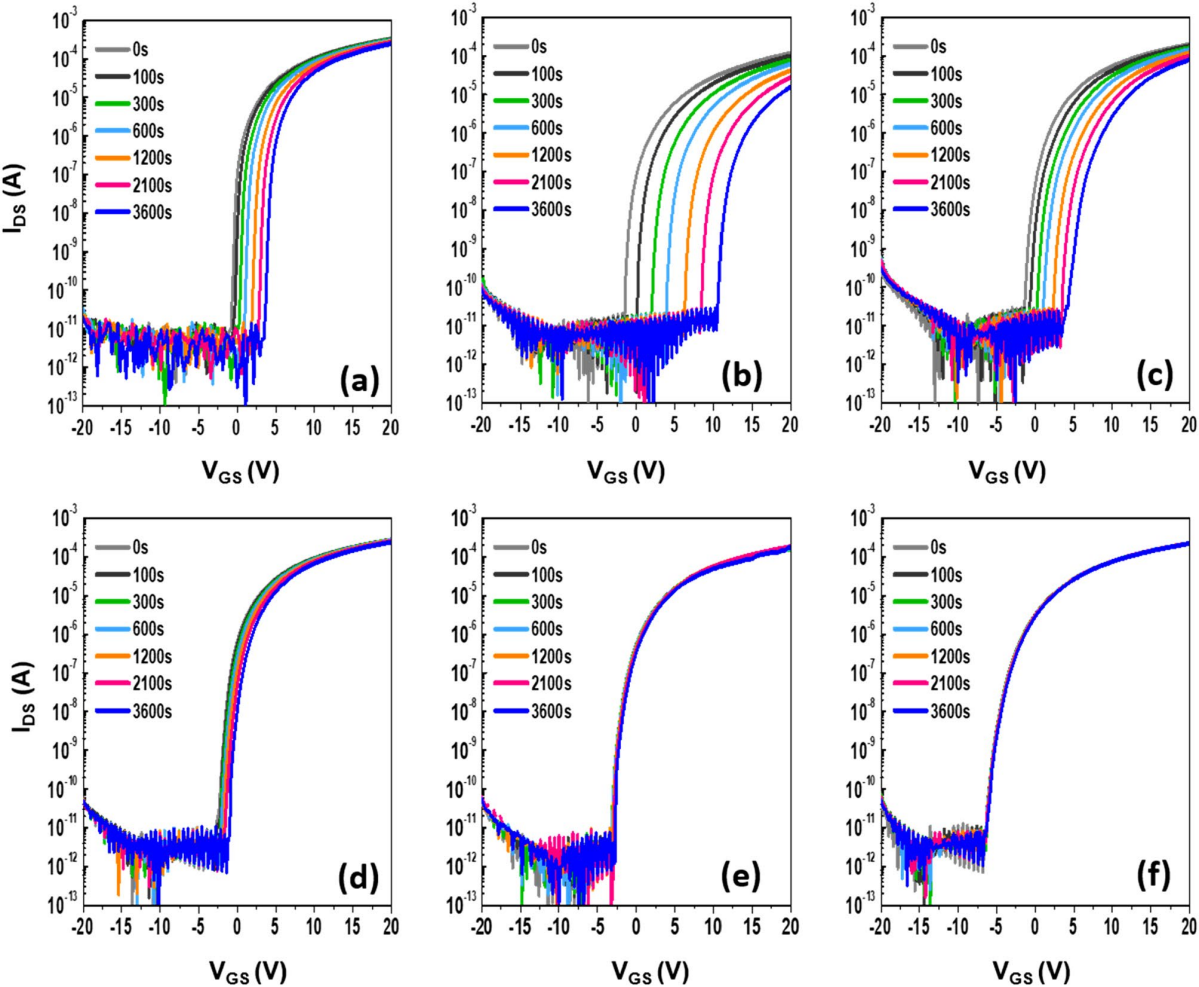
Evolution of transfer characteristics of IZTO TFTs prepared with (**a**) 19 nm after annealing time of 1 h at 350 °C and the different thicknesses: (**b**) 5, (**c**) 10, (**d**) 19, (**e**) 30, and (**f**) 50 nm after an annealing time of 1 h at 700 °C under PBS conditions. The PBS stress conditions are *V*_*GS*_ = *V*_*TH*_ + 20 V and *V*_*DS*_ = 5.1 V.

**Figure 11:**
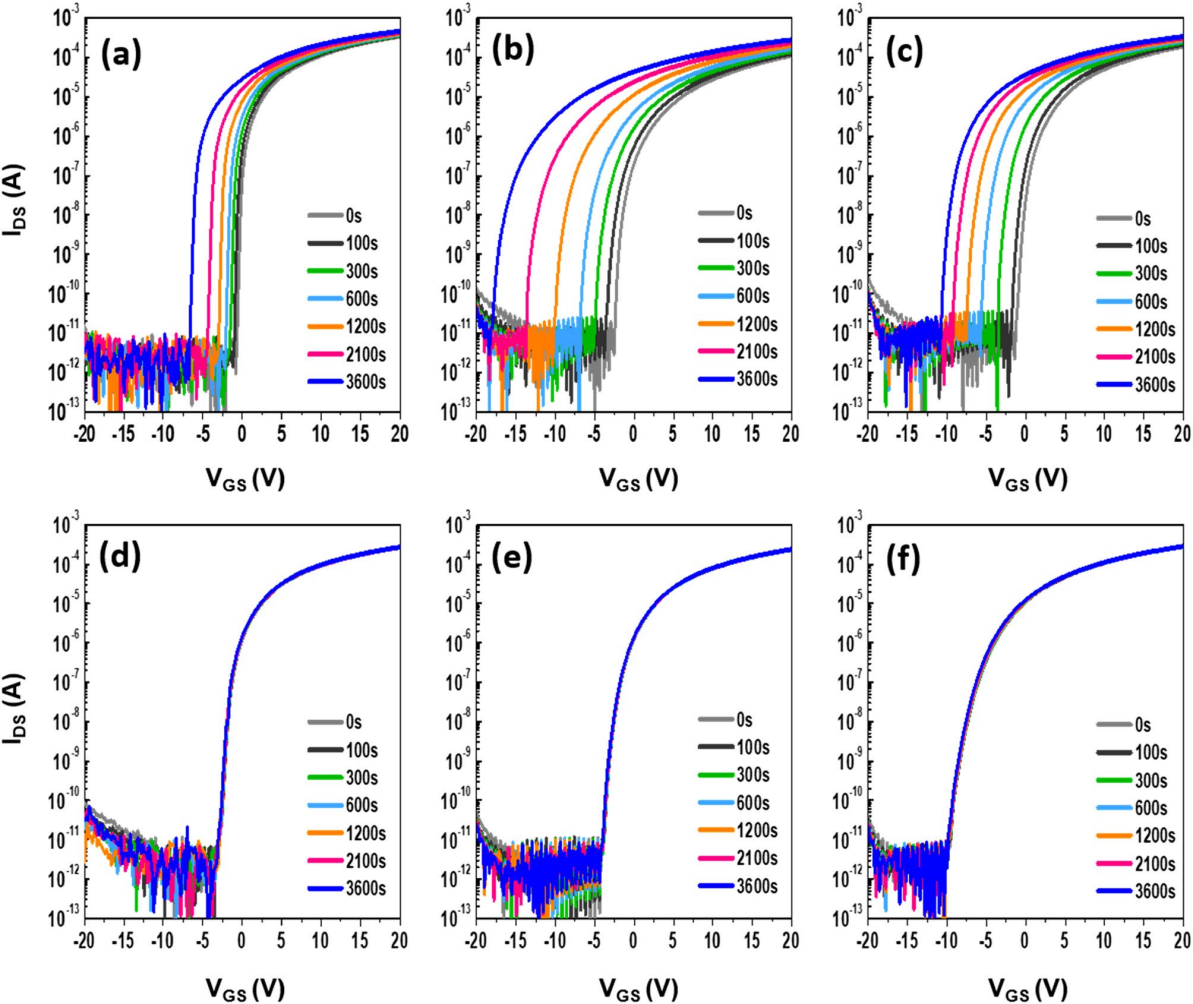
Evolution of transfer characteristics of IZTO TFTs prepared with (**a**) 19 nm film after annealing for 1 h at 350 °C and different thicknesses: (**b**) 5, (**c**) 10, (**d**) 19, (**e**) 30, and (**f**) 50 nm after an annealing time of 1 h at 700 °C under NBS conditions. The NBS stress conditions are *V*_*GS*_ = *V*_*TH*_—20 V and *V*_*DS*_ = 5.1 V.

The gate bias stress instability, which is a critical figure-of-merit for integrated circuitry applications, was examined for the given set of IZTO FETs. Figure 10 shows variations in the *I*_*DS*_–*V*_*GS*_ transfer characteristics of the IZTO FETs under a positive gate bias stress (PBS) as a function of the stress time up to 3600 s. The devices were stressed under a gate bias of (*V*_*TH*_ + 20) V and a drain bias of 5.1 V. The device with an *a*-IZTO channel layer at *T*_*A*_ = 350 °C (*t*_*ch*_ = 19 nm, annealing time of 1 h) was tested for comparison, as shown in Fig. 10a. The *∆V*_*TH*_ value for the *a*-IZTO device was 4.4 V after the PBS duration. The FETs with an amorphous 5-nm-thick IZTO channel layer at *T*_*A*_ = 700 °C suffered from a huge positive *V*_*TH*_ shift (*∆V*_*TH*_ =  + 12.2 V) after the identical PBS duration (Fig. 10b). This excessive deterioration is related to the fact that it showed the largest *N*_*T*_, as previously mentioned. The adverse creation of *N*_*T*_ is observed when the oxide channel thickness for FETs was ultrathin (≤ 5 nm) due to the surface effects^60^. In addition, the PBS-induced enhanced absorption of oxygen gas due to the unencapsulated nature of the device can aggravate the *V*_*TH*_ shift. In the case of thinner channel devices where the screening length is larger than the channel thickness, the newly adsorbed oxygen source on the IZTO can act as an acceptor-like trap site, leading to the huge positive *V*_*TH*_ shift under PBS^22,61^. As the channel thickness increased, the PBS stability of the FETs improved. The *V*_*TH*_ shifts for FETs with channel thicknesses of 19, 30, and 50 nm were reduced to + 1.52, + 0.37 and + 0.23 V, respectively, as shown in Fig. 10d,e and f. These values were smaller than that (+ 4.4 V) of FETs with an *a*-IZTO channel layer at *T*_*A*_ = 350 °C. This superior PBS stability should be attributed to the crystallization effect. Bi-stable centers such as the bond length/angle spread, under-saturated coordination, and dangling bonds in *a*-IZTO substance are eliminated as a result of lattice ordering, which results in strong immunity against external PBS. Similar behavior was also observed for the NBS-induced instabilities of the given set of IZTO FETs (Fig. 11). The devices were stressed under a gate bias of (*V*_*TH*_—20) V and a drain bias of 5.1 V. The control *a*-IZTO FETs at *T*_*A*_ = 350 °C showed a *∆V*_*TH*_ of 5.90 V after NBS duration (Fig. 11a). The NBS-induced *∆V*_*TH*_ values for devices with 5 and 10-nm-thick IZTO at *T*_*A*_ = 700 °C increased to − 14.8 and − 9.5 V, respectively (Fig. 11b,c). The smallest *∆V*_*TH*_ value was achieved for the FETs with a 19-nm-thick fully crystallized channel layer at *T*_*A*_ = 700 °C (see Fig. 11d).

### Carrier condition mechanism

**Figure 12:**
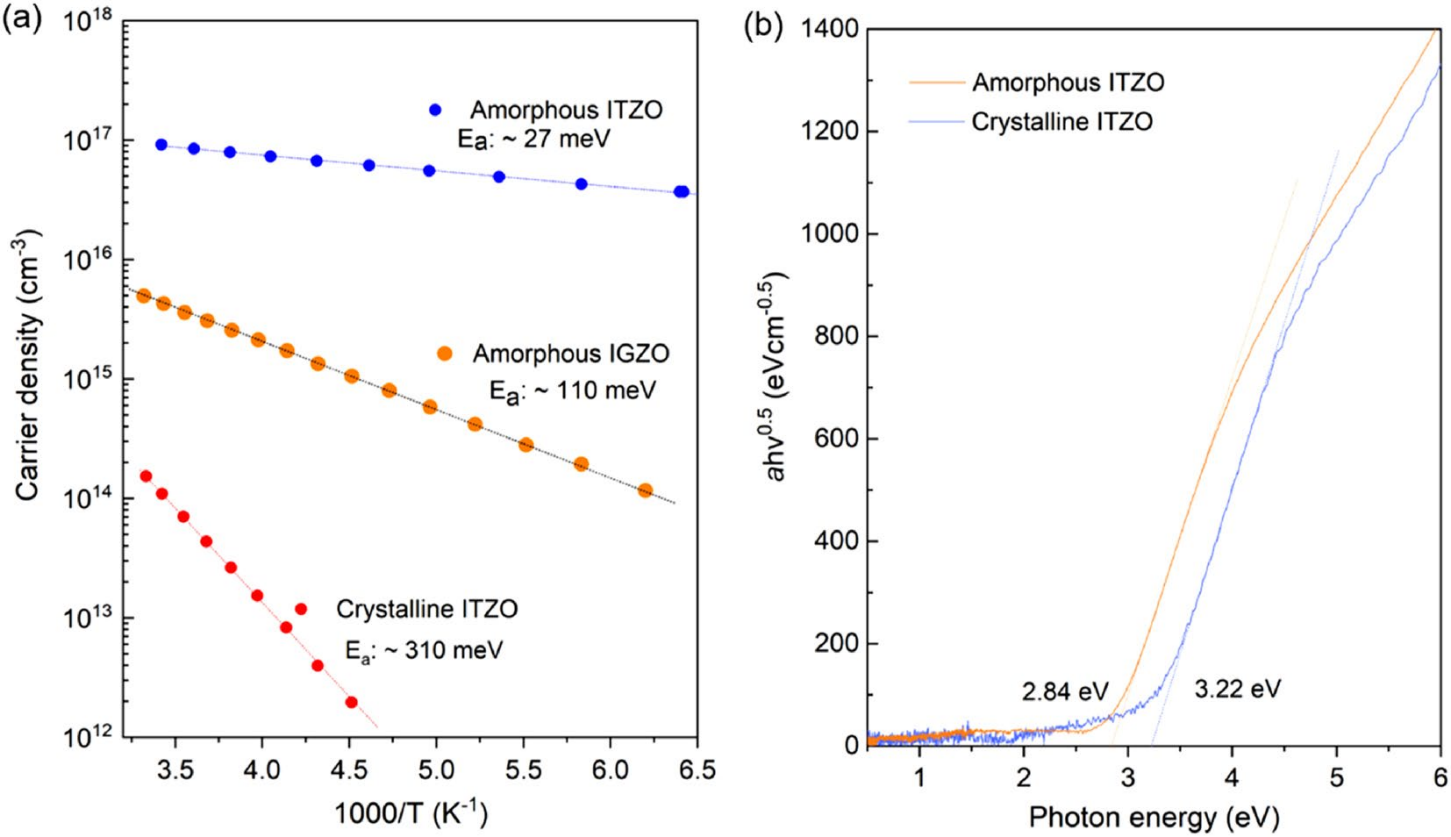
(**a**) Temperature dependent Hall measurement result of *N*_*e*_*.* (**b**) Comparison of optical *E*_*GS*_ between amorphous and crystalline IZTO thin films.

Temperature dependent Hall measurements were conducted to further study the intrinsic electrical properties of crystalline IZTO. As shown in Fig. 8, the crystalline IZTO possesses superior electrical properties compared to amorphous IZTO. The activation energy (*E*_*a*_) of the carrier density in IZTO films was extracted from the relation of *n*_*e*_ = *N*_*0*_ exp(− *E*_*a*_/*kT*). As seen in Fig. 12a, a large *E*_*a*_ of ~ 0.31 eV was confirmed for crystalline IZTO, whereas very small *E*_*a*_ values of 0.027 eV and 0.11 eV were obtained for the *a*-IZTO and *a*-IGZO, respectively. The hydrogen in amorphous transparent oxide semiconductors acts as a shallow donor. Certainly, the hydrogen in the crystallized IZTO substance can form a deep donor responsible for the ionization energy of ~ 0.31 eV. However, this is unlikely to occur because the post-annealing at 700 °C is high enough to desorb all hydrogens. The modification of electronic structure through crystallization can provide an alternative rationale for the increased *E*_*a*_ value. The activation energy of donor levels in semiconductors should be strongly dependent on energy levels such as the conduction band minimum level (*E*_*CBM*_)^62^. To assess the optical properties for the amorphous and crystalline IZTO films, the optical band-gap values were extracted by extrapolating the best fit line in the plot of (*αhν*)^0.5^ versus *hν* to the x-axis intercept for the IZTO films (Fig. 12b). The optical band gap (*E*_*G*_) increased from 2.84 eV (*a*-IZTO film) to 3.22 eV due to the crystallization at 700 °C, which means that either *E*_*CBM*_ or *E*_*VBM*_ changed by 0.35 eV. For IGZO, the transformation from the amorphous phase to the crystalline phase widens *E*_*G*_ and moves *E*_*CBM*_ toward the vacuum energy level^63^. The local structures between amorphous and crystalline IGZO phases were responsible for this difference. Less strained crystalline phase structures compared to the amorphous IGZO phase involves a smaller overlap between the metal cations s-orbitals^64^. Considering the similarities between IGZO and IZTO, the deep activation behavior of the crystalline IZTO film can be attributed to the *E*_*G*_ widening-induced *E*_*CBM*_ lifting effect. That is, the local structure such as coordination numbers should be different for the amorphous and crystalline phase. For crystalline phase, coordination number tends to increase compared to amorphous phase. Even if the similar point defect is generated, the local electrostatic charge will be different depending on the local coordinate structures. It may constitute the reason why the crystallize IZTO also possess the different *E*_*A*_ with amorphous phase. On the other hand, the relatively large *E*_*a*_ of donor levels is responsible for their stability against NBS and PBS as compared with *a*-IZTO.

## Discussion

N-type IZTO thin films with different thicknesses ranging from 5 to 50 nm were annealed at 350 and 700 °C to investigate the feasibility of using polycrystalline oxide phases in FET applications. Though the amorphous phase was obtained at *T*_*A*_ = 350 °C, annealing at *T*_*A*_ = 700 °C resulted in various phases including amorphous, weakly crystalline and strongly crystalline phases depending on the film thickness. A reasonably high carrier mobility (35.9 cm^2^/Vs) was obtained in FETs with amorphous 19-nm-thick IZTO at *T*_*A*_ = 350 °C. However, these devices fabricated at low temperature of 350 °C suffered from non-negligible instabilities with *∆V*_*TH*_ values under PBS and NBS of 4.4 V and − 5.9 V, respectively. Complete crystallization occurred when the 19-nm-thick IZTO film was treated at the elevated annealing temperature of 700 °C, as characterized by the highly aligned bixbyite crystal and spinel structure. The resulting crystalline IZTO FETs exhibited superior performance and PBS/NBS stability compared to their counterpart *a*-IZTO FETs: the *µ*_*FE*_ value and *I*_*ON/OFF*_ ratio were improved to 39.2 cm^2^V^−1^ s^−1^ and 9.7 × 10^8^, respectively. Also, PBS and NBS-induced *∆V*_*TH*_ values for the crystalline IZTO FETs diminished to + 1.52 and − 0.13, respectively. This was attributed to the formation of highly ordered cubic crystal structures showing the smallest *N*_*T*_. However, when the channel thickness was ≥ 30 nm, the *V*_*TH*_ value for FETs with the fully crystallized IZTO at *T*_*A*_ = 700 °C shifted in the negative direction and was accompanied by *V*_*TH*_ non-uniformity. The optimal thickness for the polycrystalline IZTO transistors should be carefully designed because the normally-on operation is undesirable in terms of low standby power consumption. From this investigation, we concluded that the poly-crystallization approach enables high performance IZTO transistors with excellent stability though the *GB* defects inevitably created as a result of crystallization.

## Methods

### Materials and device fabrication

Amorphous IZTO thin films ranging from 5 to 50 nm were deposited on the SiO_2_/Si substrate using rf magnetron sputtering at room temperature. The 3-inch sputtering IZTO target consisted of an indium oxide (In_2_O_3_), zinc oxide (ZnO), and tin oxide (SnO_2_) compound with a molar ratio of 1: 4: 4 (cation atomic percentage of In: Zn: Sn is 20: 40: 40). The rf power and working pressure during sputtering were fixed to 50 W and 3 mtorr under an Ar atmosphere, respectively. The as-deposited IZTO films were subjected to thermal treatment in an ambient atmosphere at different temperatures (350, 600, 650, and 700 °C) and times (1, 2, and 4 h). Device performance of semiconducting IZTO films was evaluated by fabricating the bottom-gate thin-film transistors. A heavily doped (< 0.005 Ω cm) Si substrate and a thermally-grown 100-nm-thick SiO_2_ layer served as the gate electrode and gate dielectric, respectively. After depositing the IZTO film on SiO_2_/Si, a tin-doped indium oxide (ITO) film as a source/drain (*S*/*D*) electrode was deposited using the identical sputtering system. All the active channel and *S*/*D* electrode layers were patterned through a shadow mask during each deposition. The resulting FETs had a channel width (*W*) of 1000 μm and a length (*L*) of 300 μm. Then, the fabricated FETs were subjected to thermal annealing in an ambient atmosphere at different annealing temperatures (350, 600, 650, and 700 °C) and times (1, 2, and 4 h).

### Film and device characterization

The structural properties of the IZTO films were analyzed by conventional grazing-incidence X-ray diffraction (GIXD, Smart Lab, Regaku, Japan) using Cu *K*α radiation (*λ* = 1.54056 Å). Also, synchrotron-based high-resolution two-dimensional (2D) GIXD measurements were performed on the IZTO films at the Pohang Accelerator Laboratory, 6D and 9A beamlines^65^. Each sample was mounted on a two-axis goniometer on top of an x–z stage, and the scattering intensity was measured using a 2D Mar CCD detector. The physical thickness of the IZTO films was calculated from the oscillation scattering period (*ΔQ*_z_) of the X-ray reflectivity profiles using the following equation: *∆Q*_z_ = 2π/*t*_ch_, where *t*_ch_ is the thickness of the IZTO films. The thickness of the IZTO films was also double-checked by spectroscopic ellipsometry (SE, Elli-SE, Ellipso Technology Co.). The microstructure of the IZTO films on Si/SiO_2_ substrates was analyzed by both field emission scanning electron microscopy (FE-SEM, Verios G4 UC, FEI Co.) and atomic force microscopy (AFM, Multimode 8, Bruker). The cross-sectional nanoscale structures for IZTO films were observed using Cs corrected transmission electron microscopy (CS-TEM, JEM 2100F, JEOL Ltd). The TEM samples were prepared using a focused ion beam-field scanning electron microscope (FIB-FESEM, Helios G4, Thermo Fisher Scientific Co.) employing a Ga liquid metal ion source at an acceleration voltage of 30 kV. The elemental depth profiles from the IZTO to the SiO_2_ were measured by the time-of-flight secondary ion mass spectrometry (TOF–SIMS, ION-TOF). The chemical composition of the IZTO films was determined by X-ray fluorescence (XRF, ZSX Primus II, Rigaku) spectroscopy, for which the atomic concentration was calibrated by proton-induced X-ray emission. The cation compositions of In: Zn: Sn in as-deposited and 350 °C-annealed IZTO films were 24–25: 37–38: 38–39 at%, as determined by XRF, which was double-checked by EDS analysis. The different compositions of IZTO films compared to that of the IZTO sputtering target are due to the different sputtering yields of constituent cation atoms. The cation composition of In: Zn: Sn in the IZTO films annealed at 700 °C with the different physical thicknesses of 10, 19, and 50 nm were 23–24: 35–37: 40–41 at%. The slight lower fraction (35–37 at%) of Zn in the 700 °C-annealed IZTO films suggests that it was lost during high temperature annealing due to its volatile nature. Optical band gaps of IZTO films were estimated from optical absorption spectra (UH4150, Hitachi). The Hall mobility (*μ*_Hall_) and free carrier concentration (*N*_*e*_) of the IZTO films were evaluated from Hall effect measurements using the van der Pauw configuration. Temperature dependent Hall measurements were carried out using an AC field Hall effect measurement apparatus (ResiTest8400, Toyo Corp). The 19-nm-thick *a*-IZTO and *c*-IZTO films were deposited on Si/SiO_2_ substrates, which was subjected to the thermal annealing at 350 and 700 °C, respectively. For comparison, *a*-IGZO films annealed at 350 °C in an ambient atmosphere for 1 h were also characterized where the cation atomic percentage of In: Ga: Zn was 1: 1: 1. Then, the ITO electrode with 180 nm thickness on the IZTO and IGZO samples was sputtered through shadow mask, which was followed by the contact annealing 300 °C for 1 h in an air atmosphere for Ohmic contact. Electrical characteristics of the IZTO FETs were measured at room temperature under a dark ambient condition using a Keithley 4200 analyzer. The field-effect mobility (*μ*_FE_) for the given FETs was calculated based on the maximum peak value at a drain voltage (*V*_DS_) of 0.1 V. The threshold voltage (*V*_TH_) was determined as the gate voltage (*V*_GS_) that induced a drain current (*I*_DS_) of *L*/*W* × 10 nA at a *V*_DS_ of 5.1 V. The subthreshold gate swing (*SS* = d*V*_GS_ / dlog *I*_DS_) was extracted from the linear part of the log(*I*_DS_) − *V*_GS_ plot.

## Supplementary information

Supplementary Information

## References

[1] Nomura K (2014). Room-temperature fabrication of transparent flexible thin-film transistors using amorphous oxide semiconductors. Nature.

[2] Kamiya T (2010). Present status of amorphous In–Ga–Zn–O thin-film transistors. Sci. Technol. Adv. Mater..

[3] Jang HJ (2019). Progress of display performance: AR, VR, QLED, OLED, and TFT. J. Inf. Disp..

[4] Fortunato E (2012). Oxide semiconductor thin-film transistors: a review of recent. Advances Adv. Mater..

[5] Kwon JY, Jeong JK (2015). Recent progress in high performance and reliable n-type transition metal oxide-based thin film transistors. Semicond. Sci. Technol..

[6] Park J-S (2014). Overview of electroceramic materials for oxide semiconductor thin film transistors. J. Electroceram..

[7] Noh J-Y (2013). Cation composition effects on electronic structures of In–Sn–Zn–O amorphous semiconducting. J. Appl. Phys..

[8] Song JH (2014). Achieving high field-effect mobility exceeding 50 cm^2^/Vs in In–Zn–Sn–O thin-film transistors. IEEE Electron Device Lett..

[9] Ryu MK (2009). High performance thin film transistor with cosputtered amorphous Zn–In–Sn–O channel: combinatorial approach. Appl. Phys. Lett..

[10] Hwang ES (2018). In_2_Ga_2_ZnO_7_ oxide semiconductor based charge trap device for NAND flash memory. Nanotechnology.

[11] Rha SH (2012). Vertically integrated submicron amorphous-In_2_Ga_2_ZnO_7_ thin film transistor using a low temperature process. Appl. Phys. Lett..

[12] Choi S (2019). A novel structure for improving erase performance of vertical channel NAND flash with an indium–gallium–zinc-oxide channel. IEEE Trans. Electron Devices..

[13] Ahn M-J, Cho W-J (2016). Transparent multi-level-cell nonvolatile memory with dual-gate amorphous indium–gallium–zinc oxide thin-film transistors. Appl. Phys. Lett..

[14] Rha SH (2013). Double-layered vertically integrated amorphous-In_2_Ga_2_ZnO_7_ thin-film transistor. Appl. Phys. Lett..

[15] Jung JS (2012). The charge trapping characteristics of SiN_4_ and Al_2_O_3_ layer on amorphous–indium–gallium–zinc oxide thin films for memory application. Appl. Phys. Lett..

[16] Chang S (2008). Efficient suppression of charge trapping in ZnO-based transparent thin film transistors with novel Al_2_O_3_/HfO_2_/Al_2_O_3_ structure. Appl. Phys. Lett..

[17] Na S-Y, Yoon S-M (2019). Impacts of HfO_2_/ZnO stack-structured charge-trap layers controlled by atomic layer deposition on nonvolatile memory characteristics of In–Ga–Zn–O channel charge-trap memory thin-film transistors. IEEE J. Electron Devices Soc..

[18] Wehrsohn RB (2000). Dangling-bond defect state creation in microcrystalline silicon thin-film transistors. Appl. Phys. Lett..

[19] Karim KS (2004). Drain-bias dependence of threshold voltage stability of amorphous silicon TFTs. IEEE Electron Device Lett..

[20] Chen C-Y (2006). Negative bias temperature instability in low-temperature polycrystalline silicon thin-film transistors. IEEE Trans. Electron Devices..

[21] Son K-S (2008). 4inch QVGA AMOLED drive by the threshold voltage controlled amorphous GIZO (Ga_2_O_3_-In_2_O_3_-ZnO) TFT. SID Symp. Dig. Tech. Pap..

[22] Jeong JK (2008). Origin of threshold voltage instability in indium-gallium-zinc oxide thin film transistors. Appl. Phys. Lett..

[23] Park J-S (2008). Control of threshold voltage in ZnO-based oxide thin film transistors. Appl. Phys. Lett..

[24] Kang Y (2015). Hydrogen bistability as the origin of photo-bias-thermal instabilities in amorphous oxide semiconductors. Adv. Electron. Mater..

[25] Nomura K (2004). Carrier Transport in transparent oxide semiconductor with intrinsic structural randomness probed using single-crystalline InGaO_3_(ZnO)_5_ films. Appl. Phys. Lett..

[26] Nomura K (2006). Amorphous oxide semiconductors for high-performance flexible thin-film transistors. Jpn. J. Appl. Phys..

[27] Ahn BK (2011). Origin of device performance degradation in InGaZnO thin-film transistors after crystallization. Jpn. J. Appl. Phys..

[28] Kim GH (2009). Formation mechanism of solution-processed nanocrystalline InGaZnO thin film as active channel layer in thin-film transistor. J. Electrochem. Soc..

[29] Park K (2015). Reliability of crystalline indium–gallium–zinc-oxide thin-film transistors under bias stress with light illumination. IEEE Trans. Electron Devices..

[30] Shin Y (2017). The mobility enhancement of indium gallium zinc oxide transistors via low-temperature crystalline using a tantalum catalytic layer. Sci. Rep..

[31] Baccarani G (1978). Transport properties of polycrystalline silicon films. J. Appl. Phys..

[32] Kimura M (2001). Dependence of polycrystalline silicon thin-film transistor characteristics on the grain-boundary location. J. Appl. Phys..

[33] Kimura M (2001). Device simulation of carrier transport through grain boundaries in lightly doped polysilicon films and dependence on dopant density. Jpn. J. Appl. Phys..

[34] Medvedeva JE (2017). Recent advances in understanding the structure and properties of amorphous oxide semiconductors. Adv. Electron. Mater..

[35] Yabuta H (2014). Microscopic structure and electrical transport property of sputtered-deposited amorphous indium–gallium–zinc oxide semiconductor films. J. Phys. Conf. Ser..

[36] Buchholz DB (2014). The structure and properties of amorphous indium oxide. Chem. Mater..

[37] Kim H (2017). Impact of bias stability for crystalline InZnO thin-film transistors. Appl. Phys. Lett..

[38] Chang KJ (1988). Theory of hydrogen passivation of shallow-level dopants in crystalline silicon. Phys. Rev. Lett..

[39] Bang J (2008). Diffusion and thermal stability of hydrogen in ZnO. Appl. Phys. Lett..

[40] Bang J (2017). Hydrogen anion and subgap states in amorphous In–Ga–Zn–O thin films for TFT applications. Appl. Phys. Lett..

[41] Hanyu Y (2013). Hydrogen passivation of electron trap in amorphous In–Ga—Zn–O thin-film transistors. Appl. Phys. Lett..

[42] On N (2017). Origin of electrical instabilities in self-aligned amorphous In–Ga–Zn–O thin-film transistors. IEEE Trans. Electron Devices.

[43] Sadananda Kumar N (2014). Effect of annealing on the properties of zinc oxide nanofiber thin films grown by spray pyrolysis technique. Appl. Nanosci..

[44] Shi L (2008). Annealing temperature effects on photoelectrochemical performance of bismuth vanadate thin film photoelectrodes. RSC. Adv..

[45] Ahmed NM (2019). The effect of post annealing temperature on grain size of indium-tin-oxide for optical and electrical properties improvement. Res. in Phys..

[46] Wang J (2007). One-pot hydrothermal synthesis of highly efficient SnO_x_/Zn_2_SnO_4_ composite photocatalyst for the degradation of methyl orange and gaseous benzene. Appl. Catal. B: Environ..

[47] Yuan H-L, Li J-C (2017). Effect of annealing temperature on the growth of Zn–Sn–O nanocomposite thin films. J. Alloys Compd..

[48] Nayak AK (2015). Biomolecule-assisted synthesis of In(OH)_3_ nanocubes and In_2_O_3_ nanoparticles: photocatalytic degradation of organic contaminants and CO oxidation. Nanotechnology.

[49] Liu D (2008). High-pressure x-ray diffraction and raman spectra study of indium oxide. J. Appl. Phys..

[50] Harvey SP (2008). Subsolidus phase relationships in the ZnO–In_2_O_3_–SnO_2_ system. J. Am. Ceram. Soc..

[51] Hoel CA (2010). Transparent conducting oxides in the ZnO–In_2_O_3_–SnO_2_ system. Chem. Mater..

[52] Jantzen T (2018). Thermodynamic assessment of oxide system In_2_O_3_–SnO_2_–ZnO. Chim. Techno Acta..

[53] Hoel CA (2011). Evidence for tetrahedral zinc in amorphous In_2-2x_Zn_x_Sn_x_O_3_ (a-ZITO). Z. Anorg. Allg. Chem..

[54] Hoel CA (2010). High-pressure synthesis and local structure of corundum-type In_2__–__2x_Zn_x_Sn_x_O_3_ (x ≤ 0.7). J. Am. Chem. Soc..

[55] Choi IM (2020). Achieving high mobility and excellent stability in amorphous In–Ga–Zn–Sn–O thin-film transistors. IEEE Trans. Electron Devices.

[56] Chang KM (2003). Electrical characteristics of low temperature polysilicon TFT with a novel TEOS/oxynitride stack gate dielectric. IEEE Electron Device Lett..

[57] Watanabe H (2007). Statistics of grain boundaries in polysilicon. IEEE Trans. Electron Devices..

[58] Nakata M (2013). Influence of oxide semiconductor thickness on thin-film transistor characteristics. Jpn. J. Appl. Phys..

[59] Zhang J (2015). C-Axis oriented crystalline IGZO thin-film transistors by magnetron sputtering. J. Mater. Chem. C..

[60] Lee SY (2011). Effect of channel thickness on density of states in amorphous InGaZnO thin film transistor. Appl. Phys. Lett..

[61] Park JS (2008). Electronic transport properties of amorphous indium–gallium–zinc oxide semiconductor upon exposure to water. Appl. Phys. Lett..

[62] Robertson J (2012). Properties and doping limits of amorphous oxide semiconductors. J. Non-Cryst. Solids..

[63] Kang Y (2014). Nature of visible-light absorption in amorphous semiconducting oxides. APL Mater..

[64] Kim J (2019). Ultra-wide bandgap amorphous oxide semiconductors for NBIS-free thin-film transistors. APL Mater..

[65] Gao CY (2019). Spontaneous phase separation of poly(3-hexylthiophene)s with different regioregularity for a stretchable semiconducting film. Adv. Funct. Mater..

