# Supplementary material for: Boosting carrier mobility and stability in indium–zinc–tin oxide thin-film transistors through controlled crystallization
Source: Sci Rep. 2020 Nov 2;10:18868. doi: 10.1038/s41598-020-76046-w (PMC7606507; doi:10.1038/s41598-020-76046-w)
Supplement: Supplementary file 1 — Supplementary Information [file 41598_2020_76046_MOESM1_ESM.docx]

**Supplementary Information for**

Boosting Carrier Mobility and Stability in Indium-Zinc-Tin Oxide Thin-Film Transistors through Controlled Crystallization

Nuri On^1^, Bo Kyoung Kim^1^, Yerin Kim^2^, Eun Hyun Kim^3^, Jun Hyung Lim^3^, Hideo Hosono^4^, Junghwan Kim^4^*, Hoichang Yang^2^*, and Jae Kyeong Jeong^1^*

^1^ Department of Electronic Engineering, Hanyang University, Seoul 133-791, Republic of Korea

^2^ Department of Chemical Engineering, Inha University, Incheon 22212, South Korea

^3^ R&D Center, Samsung Display, Yongin 17113, South Korea

^4^ Materials Research Center for Element Strategy, Tokyo Institute of Technology, Yokohama 226-8503, Japan

AUTHOR EMAIL ADDRESS: J. Kim ([JH.KIM@mces.titech.ac.jp](mailto:JH.KIM@mces.titech.ac.jp)); H. Yang ([hcyang@inha.ac.kr](mailto:hcyang@inha.ac.kr)); J. K. Jeong ([jkjeong1@hanyang.ac.kr](mailto:jkjeong1@hanyang.ac.kr))

**
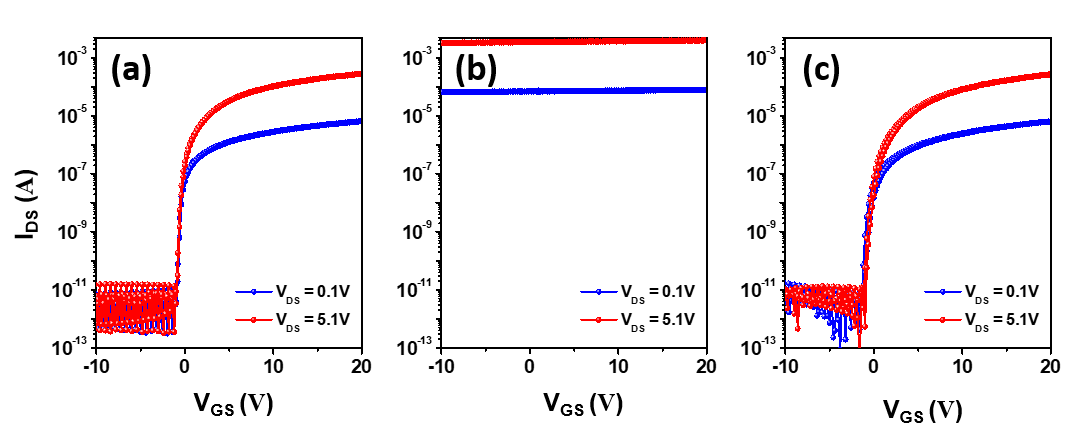
**

**Figure S1.** Transfer characteristics of the 19-nm-thick IZTO FETs annealed at different *T_A_*_S_ of (a) 350, (b) 600, and (c) 700 °C for 1 h.

**Table S1.** Summary of electrical parameters of the 19 nm IZTO FETs annealed at different *T_A_*_S_ of 350, 600, and 700 °C for 1 h.

| **Sample condition** | **350 °C** | **600 °C** | **700 °C** |
| --- | --- | --- | --- |
| *μ_FE_*  (cm^2^/Vs) | 35.9 | - | 39.7 |
| *SS*  (V/decade) | 0.24 | - | 0.26 |
| *V_TH_*  (V) | -0.41 | - | -0.21 |
| *I_ON/OFF_* | 9.8 × 10^8^ | - | 9.9 × 10^8^ |


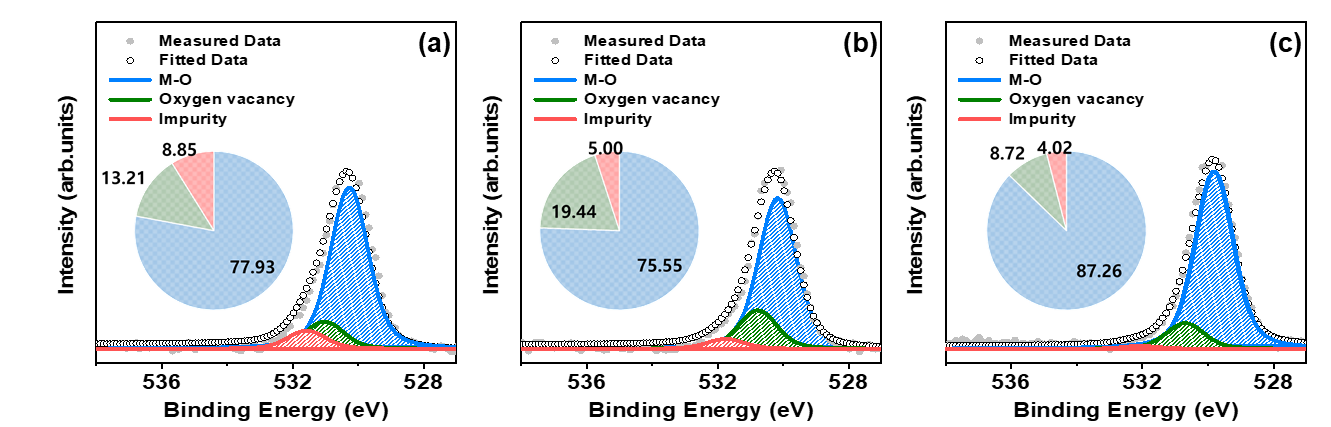


**Figure S2.** O*1s* X-ray photoelectron spectroscopy (XPS) of 19 nm IZTO thin films annealed at (a) 350 °C, (b) 600 °C and (c) 700 °C, respectively, which were obtained from depth profile XPS analysis. The O *1s* peak was deconvoluted into three subpeaks at 530.2, 530.9 and 531.9 eV. The peaks at 530.2 and 530.9 eV can be assigned to the oxygen bonded to fully coordinated metal ions and to under-coordinated metal ions. The peak at 531.9 eV was assigned as impurity-related oxygen, such as hydroxyl groups. The *V_O_* related peak area values for the IZTO films at 350 °C, 600 °C and 700 °C were 13.2, 19.4 and 8.7 %, respectively.

**
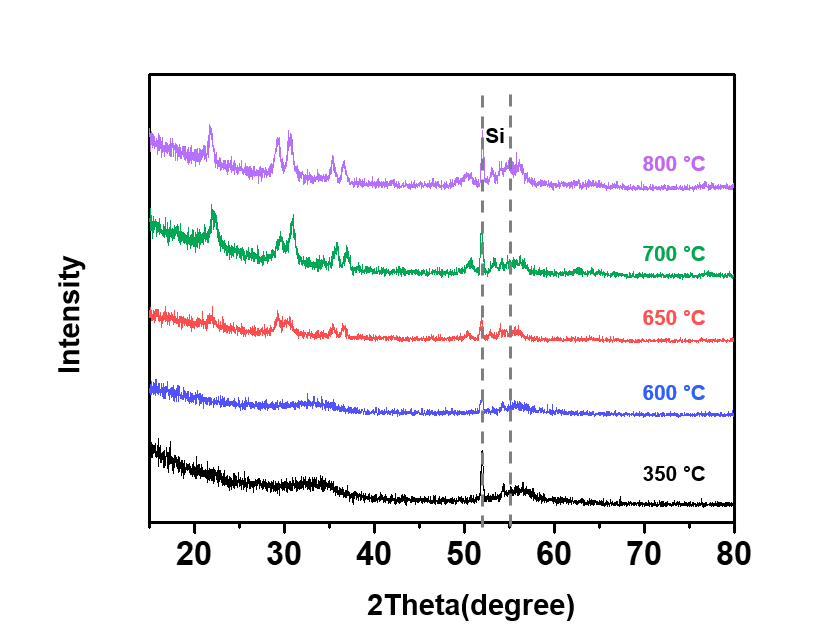
**

**Figure S3.** 1D X-ray diffraction patterns of 19-nm-thick IZTO thin films annealed at different *T_A_*_S_ for 1 h.


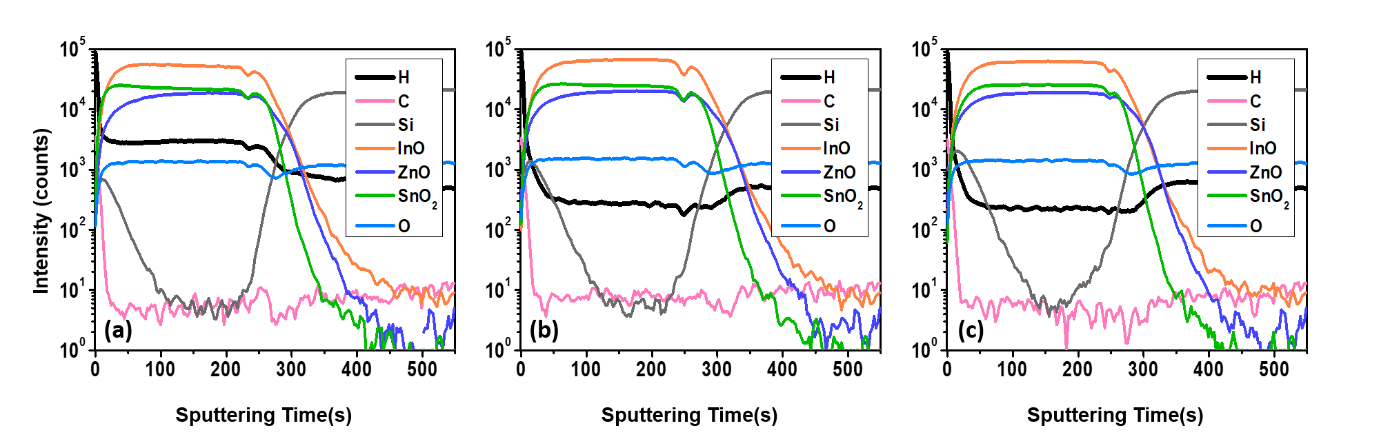


**Figure S4.** TOF-SIMS depth profile of 19-nm-thick IZTO thin films annealed at different *T_A_*_S_ of (a) 350, (b) 600, and (c) 700 °C for 1 h. Hydrogen concentration in the IZTO film decreased with increasing *T_A_* from 350 to 700 °C, indicating its rapid diffusion out.


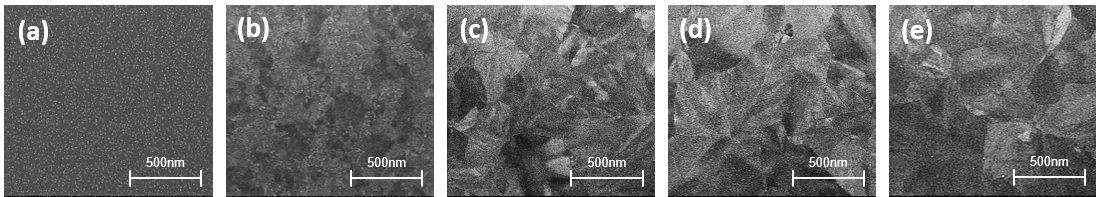


**Figure S5.** SEM morphologies of IZTO films of different thickness after annealing at 700 °C for 2 h: (a) 5 (b) 10, (c) 19, (d) 30, and (e) 50 nm.


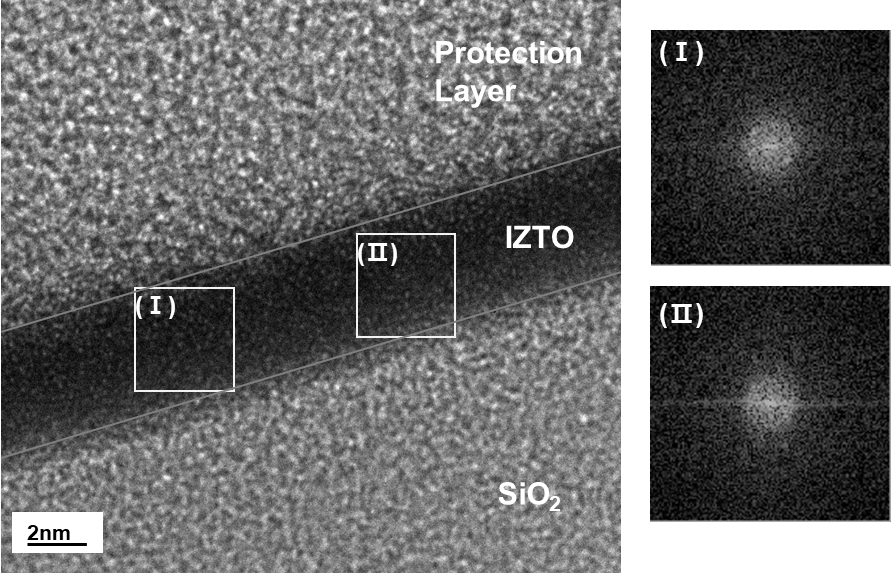


**Figure S6.** Cross-sectional TEM image of 5-nm-thick IZTO film annealed at 700 °C for 1 h on a SiO_2_/Si substrate. SADP obtained from the fast Fourier transform (FFT) at each position is given in the inset of each image.


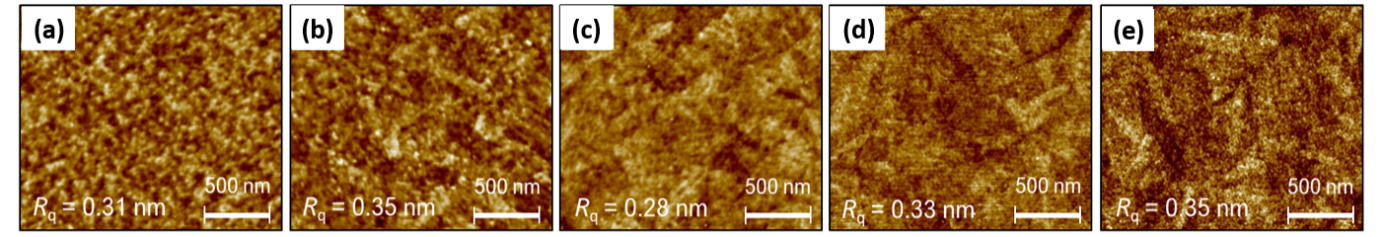


**Figure S7.** AFM topographies of IZTO films of different thickness after annealing at 700 °C for 2 h: (a) 5 (b) 10, (c) 19, (d) 30, and (e) 50 nm.


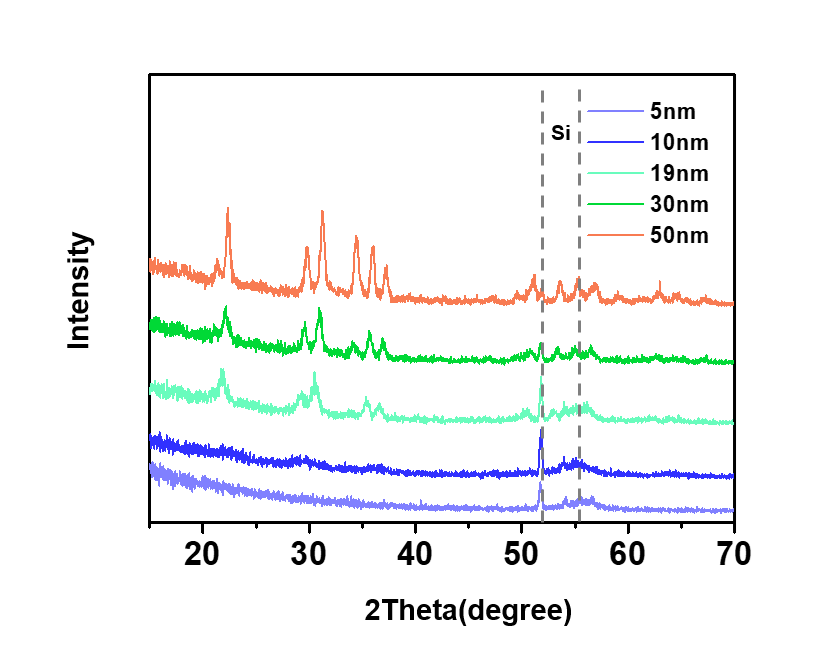


**Figure S8.** 1D X-ray diffraction patterns of IZTO thin films different thickness annealed at 700 °C for 2 h.


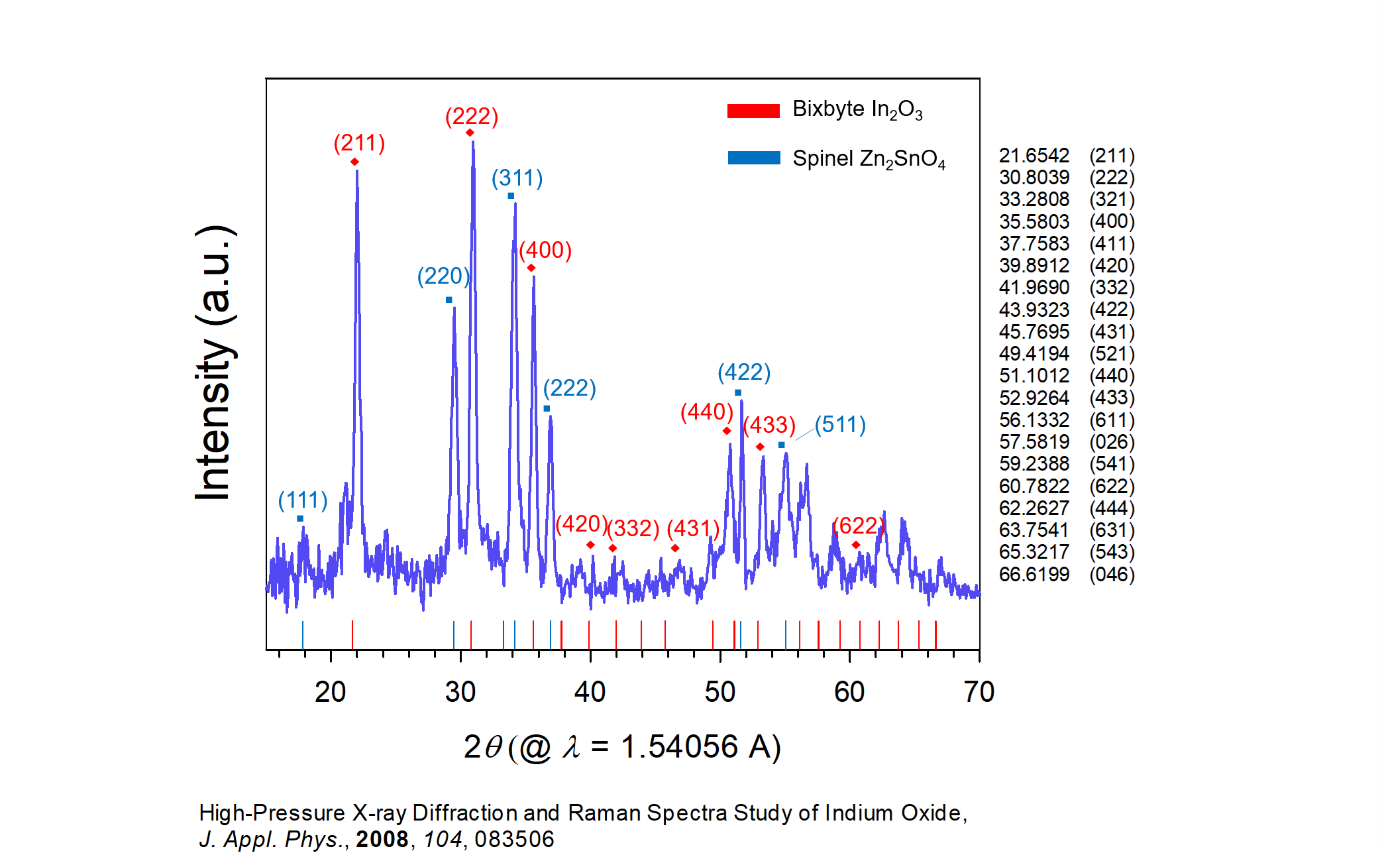


**Figure S9.** Typical X-ray diffraction pattern of 700 °C-annealed IZTO film showing typical (*hkl*) reflections in spinel Zn_2_SnO_4_ and cubic bixbyite In_2_O_3_ phases.

**Table S2.** Summary of crystal information for IZTO thin film annealed at 700 °C, as determined by the 2D GIXD patterns.

|  | **(hkl)** | **2θ (°)** | **d_hkl_ (Å)** | **Q (Å^-1^)** |
| --- | --- | --- | --- | --- |
| **In_2_O_3_** | (211) | 21.65 | 4.10 | 1.53 |
|  | (222) | 30.80 | 2.90 | 2.17 |
|  | (400) | 35.58 | 2.52 | 2.49 |
|  | (411) | 37.75 | 2.38 | 2.64 |
|  | (431) | 45.76 | 1.98 | 3.17 |
| **Zn_2_SnO_4_** | (111) | 17.86 | 4.96 | 1.27 |
|  | (220) | 29.49 | 3.02 | 2.08 |
|  | (311) | 34.20 | 2.62 | 2.40 |
|  | (222) | 36.91 | 2.43 | 2.58 |
|  | (422) | 51.65 | 1.77 | 3.56 |


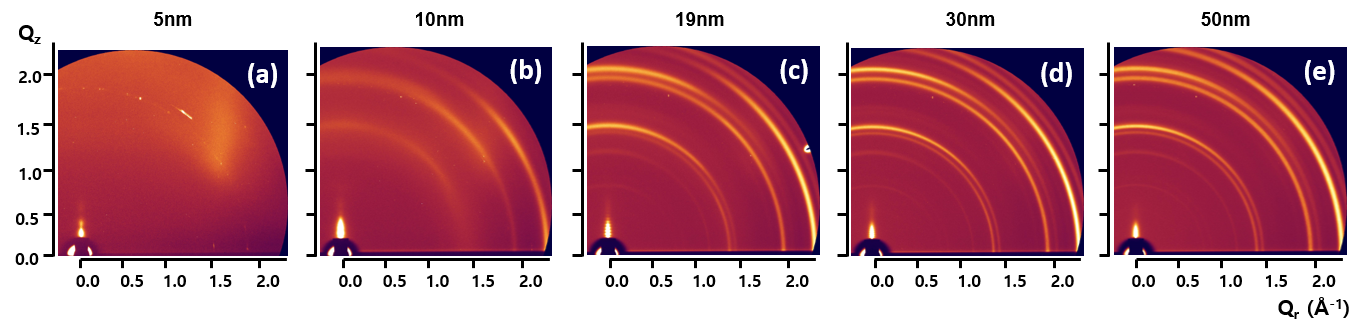
**Figure S10.** 2D GIXD patterns of IZTO films of different thickness after annealing at 700 °C for 2 h: (a) 5, (b) 10, (c) 19, (d) 30, and (e) 50 nm.


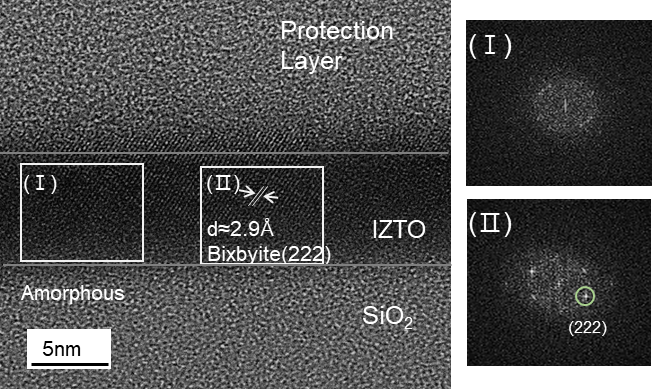


**Figure S11.** Cross-sectional TEM image of 10-nm-thick IZTO film annealed at 700 ℃ for 1 h on a SiO_2_/Si substrate. SADP obtained from the fast Fourier transform (FFT) at each position is given in the inset of each image.


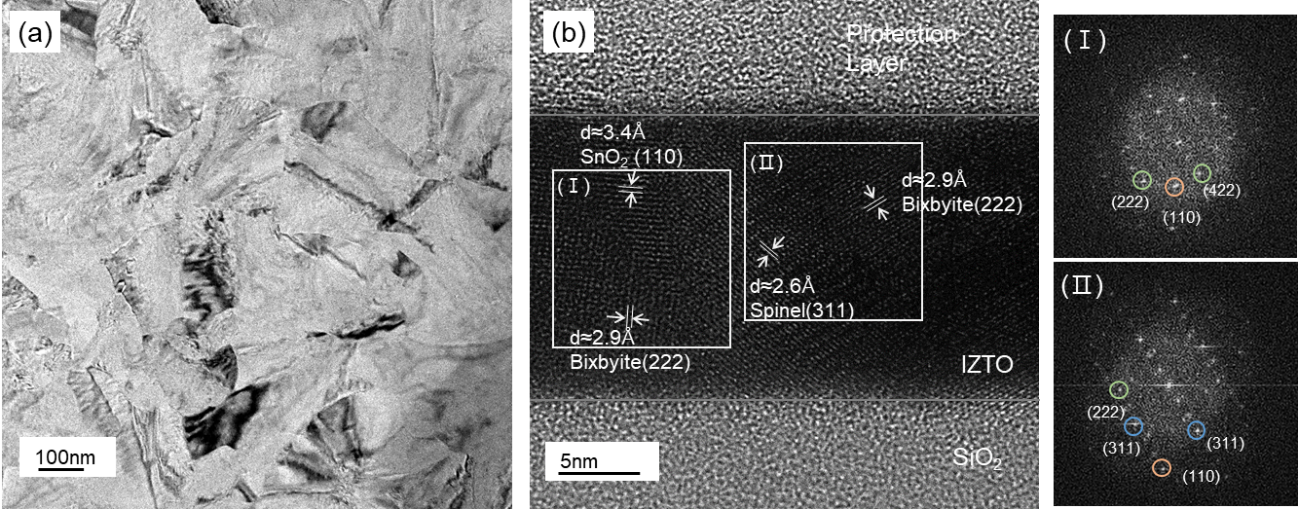


**Figure S12.** (a) Plan-view and (b) cross-sectional TEM image of 19-nm-thick IZTO film annealed at 700 ℃ for 1 h on the SiO_2_/Si substrate. SADP obtained from the FFT at each position is given in the inset in each image.


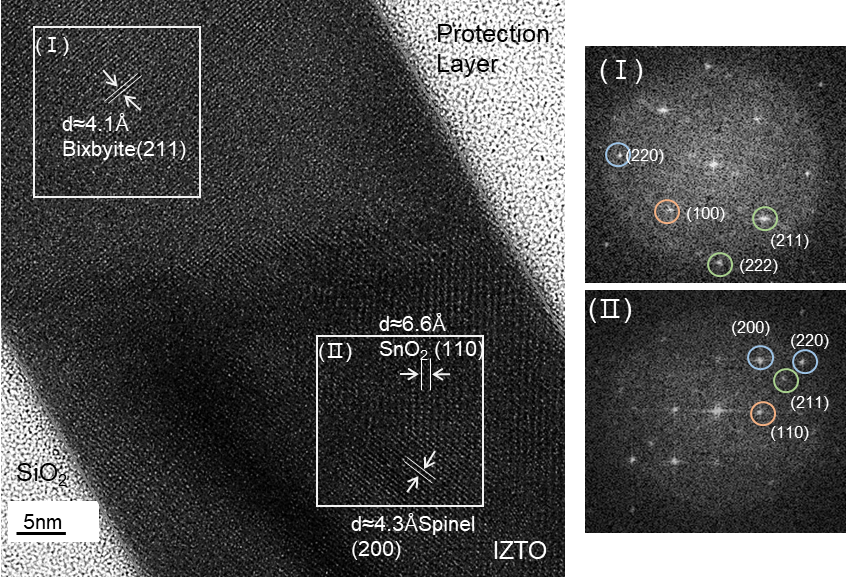


**Figure S13.** Cross-sectional TEM image of 50-nm-thick IZTO film annealed at 700℃ for 1 h on the SiO_2_/Si substrate. SADP obtained from the FFT at each position was inserted in the inset of each image.

**Table S3.** Variations in the vertical grain size for the IZTO films with IZTO films of different thickness annealed at 700 °C for 1, 2, and 4 h.

| **Sample conditions** | **Annealing time (h)** | **19-nm-thick IZTO** | **30-nm-thick IZTO** | **50-nm-thick IZTO** |
| --- | --- | --- | --- | --- |
| Vertical grain size  (nm) | 1 | 11.9 | 17.1 | 18.2 |
|  | 2 | 11.4 | 14.7 | 16.7 |
|  | 4 | 10.1 | 15.0 | 16.8 |


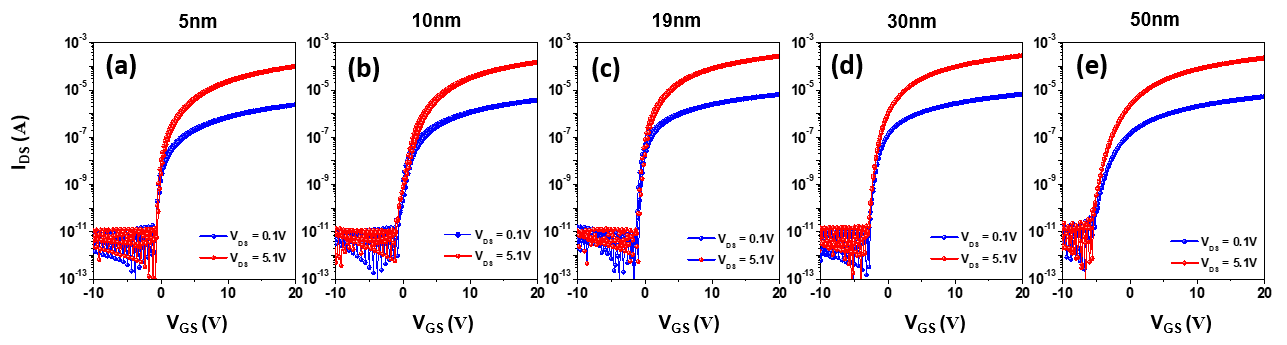


**Figure S14.** *I*_DS_-*V*_GS_ transfer curves of FETs including IZTO films of different thickness after annealing at 700 °C for 2 h: (a) 5, (b) 10, (c) 19, (d) 30, and (e) 50 nm.

**Table S4.** Typical electrical parameters of the FETs including IZTO films of different thickness annealed at 700 °C for 2 h.

| **IZTO thickness** | **Annealing time (h)** | ***µ_FE_***  **(cm^2^ V^-1^ s^-1^)** | **SS**  **(V decade^-1^)** | ***V_TH_***  **(V)** | ***I_ON/OFF_*** | ***D_it,max_***  **(cm^-2^ eV^-1^)** | ***N_T, max_***  **(cm^-3^ eV^-1^)** |
| --- | --- | --- | --- | --- | --- | --- | --- |
| 5 nm | 2 | 16.8 ± 1.9 | 0.50 ± 0.04 | 0.3 ± 0.2 | 9.8 × 10^7^ | 1.8 × 10^12^ | 3.7 × 10^18^ |
| 10 nm | 2 | 23.4 ± 1.6 | 0.55 ± 0.09 | 0.9 ± 0.3 | 4.0 × 10^8^ | 2.0 × 10^12^ | 2.0 × 10^18^ |
| 19 nm | 2 | 39.2 ± 2.4 | 0.25 ± 0.06 | -0.3 ± 0.4 | 9.9 × 10^8^ | 9.2 × 10^11^ | 4.8 × 10^17^ |
| 30 nm | 2 | 39.0 ± 3.3 | 0.46 ± 0.10 | -1.7 ± 1.5 | 9.5 × 10^8^ | 1.7 × 10^12^ | 5.6 × 10^17^ |
| 50 nm | 2 | 36.3 ± 2.0 | 0.92 ± 0.11 | -3.9 ± 2.2 | 9.8 × 10^8^ | 3.4 × 10^12^ | 6.7 × 10^17^ |


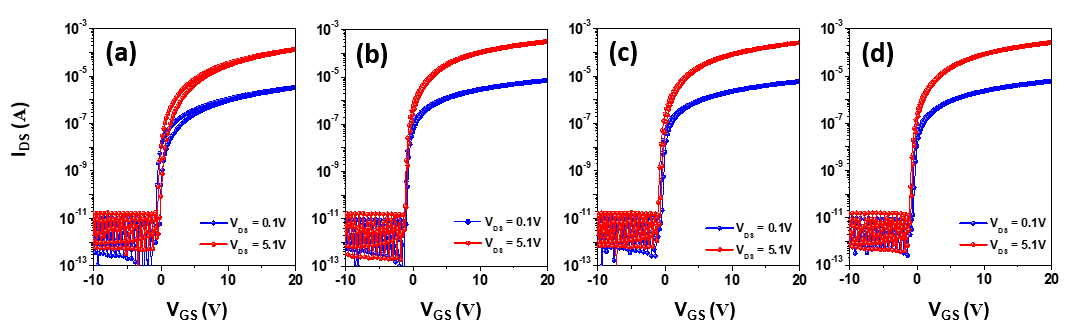


**Figure S15.** *I*_DS_-*V*_GS_ transfer curves of FETs including IZTO films of different thickness after annealing at 350 °C for 1h: (a) 5, (b) 10, (c) 30, and (d) 50 nm.

**Table S5.** Summary of electrical parameters of FETs including IZTO films of different thickness annealed at 350 °C for 1 h.

| **ID** | **5-nm-thick IZTO** | **10-nm-thick IZTO** | **30-nm-thick IZTO** | **50-nm-thick IZTO** |
| --- | --- | --- | --- | --- |
| ***µ_FE_***  **(cm^2^ V^-1^ s^-1^)** | 23.2 | 37.8 | 37.2 | 34.6 |
| ***SS***  **(V decade^-1^)** | 0.33 | 0.31 | 0.35 | 0.41 |
| ***V_TH_***  **(V)** | 0.23 | -0.76 | -0.51 | -0.56 |
| ***I_ON/OFF_*** | 9.6 × 10^7^ | 9.7 × 10^8^ | 9.3 × 10^8^ | 9.5 × 10^8^ |
